# A novel and widespread class of ketosynthase is responsible for the head-to-head condensation of two acyl moieties in bacterial pyrone biosynthesis

**DOI:** 10.3762/bjoc.11.152

**Published:** 2015-08-12

**Authors:** Darko Kresovic, Florence Schempp, Zakaria Cheikh-Ali, Helge B Bode

**Affiliations:** 1 Merck Stiftungsprofessur für Molekulare Biotechnologie, Fachbereich Biowissenschaften, Goethe Universität Frankfurt, 60438 Frankfurt am Main, Germany; 2 Buchmann Institute for Molecular Life Sciences (BMLS), Goethe Universität Frankfurt, 60438 Frankfurt am Main, Germany

**Keywords:** cell–cell communication, ketosynthase, photopyrones, pseudopyronines, quorum sensing

## Abstract

The biosynthesis of photopyrones, novel quorum sensing signals in *Photorhabdus*, has been studied by heterologous expression of the photopyrone synthase PpyS catalyzing the head-to-head condensation of two acyl moieties. The biochemical mechanism of pyrone formation has been investigated by amino acid exchange and bioinformatic analysis. Additionally, the evolutionary origin of PpyS has been studied by phylogenetic analyses also revealing homologous enzymes in *Pseudomonas* sp. GM30 responsible for the biosynthesis of pseudopyronines including a novel derivative. Moreover this novel class of ketosynthases is only distantly related to other pyrone-forming enzymes identified in the biosynthesis of the potent antibiotics myxopyronin and corallopyronin.

## Introduction

**Figure 1:**
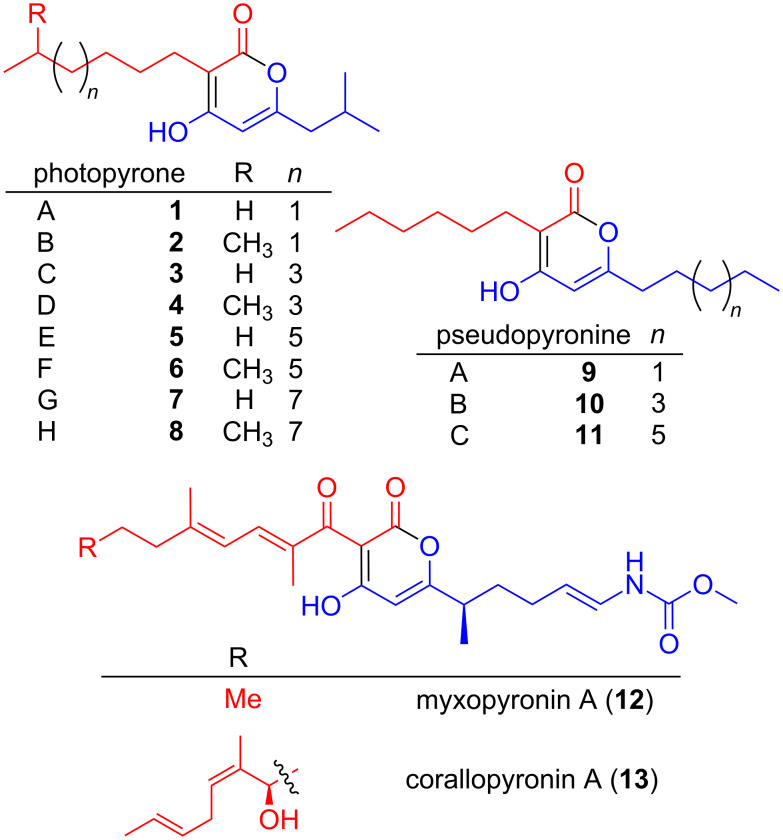
Structures of photopyrones **1–8**, pseudopyronines **9–11**, myxopyronin A (**12**) and corallopyronin A (**13**). For all α-pyrones the western (red) and the eastern (blue) acyl moiety is highlighted.

Chemical compounds containing an α-pyrone moiety are widespread in nature [1–2] and show a high diversity in their biological activity. Members of this class of compounds have been identified with antimicrobial, cytotoxic [3] and antitumor activities [4], as HIV protease inhibitors [5] and selective COX-2 inhibitors [6]. Recently, it was shown that photopyrones (Figure 1) are signaling molecules in a new cell–cell communication system in the entomopathogenic bacterium *Photorhabdus luminescens*. The system consists of endogenously produced photopyrones as signaling molecules and PluR, a LuxR-like receptor [7]. The binding of photopyrones by PluR leads to the expression of the Photorhabdus clumping factor (PCF) operon (*pcfABCDEF*) that causes clumping of cells and results in insect toxicity [7]. Responsible for the formation of photopyrones is an unusual ketosynthase, named photopyrone synthase (PpyS), which catalyzes the head-to-head condensation of two acyl moieties.

Ketosynthases (KS) are the key enzymes involved in the biosynthesis of fatty acids and polyketides [8]. They are known to catalyze C–C bond formations via a Claisen condensation between acyl- and malonylthioesters and they all share the thiolase fold [9]. The crystal structure of a mammalian [10] and a fungal [11] fatty acid synthase confirmed the dimeric nature of the KS. Recently KS have been described, whose substrates deviate from the usual ones. Examples are the widespread class of KS catalyzing the formation of 2,5-dialkylcyclohexane-1,3-diones from a β-ketoacylthioester and an α,β-unsaturated acylthioester [12]. We have recently demonstrated that dialkylresorcinols also serve as a new bacterial communication signal in the human and insect pathogen *Photorhabdus asymbiotica* [13]. A further example is OleA, which is part of the olefin biosynthesis in *Xanthomonas campestris* by promoting a head-to-head condensation of two long-chain fatty acid thioesters to form a long-chain β-ketoacid that can be further processed to an olefin [14]. KS with acylating activity have been reported in the cervimycin [15], xenocyloin [16] and nonactin biosynthesis [17].

Plasmid integration into the gene *plu4844* encoding the ketosynthase PpyS in *P. luminescens* resulted in the complete loss of photopyrone production as shown previously [7]. Additionally, heterologous expression of *ppyS*, *bkdABC* and *ngrA* in *E. coli* allowed the reconstitution of photopyrone production: Coexpression of genes encoding the branched chain α-ketoacid dehydrogenase (Bkd) complex of *P. luminescen*s (BkdABC) was required for the production of iso-fatty acids [18], whereas the activation of the acyl-carrier-protein domain within BkdB required the phosphopantetheinyl-transferase (PPtase) activity of NgrA [18].

## Results and Discussion

**Scheme 1:**
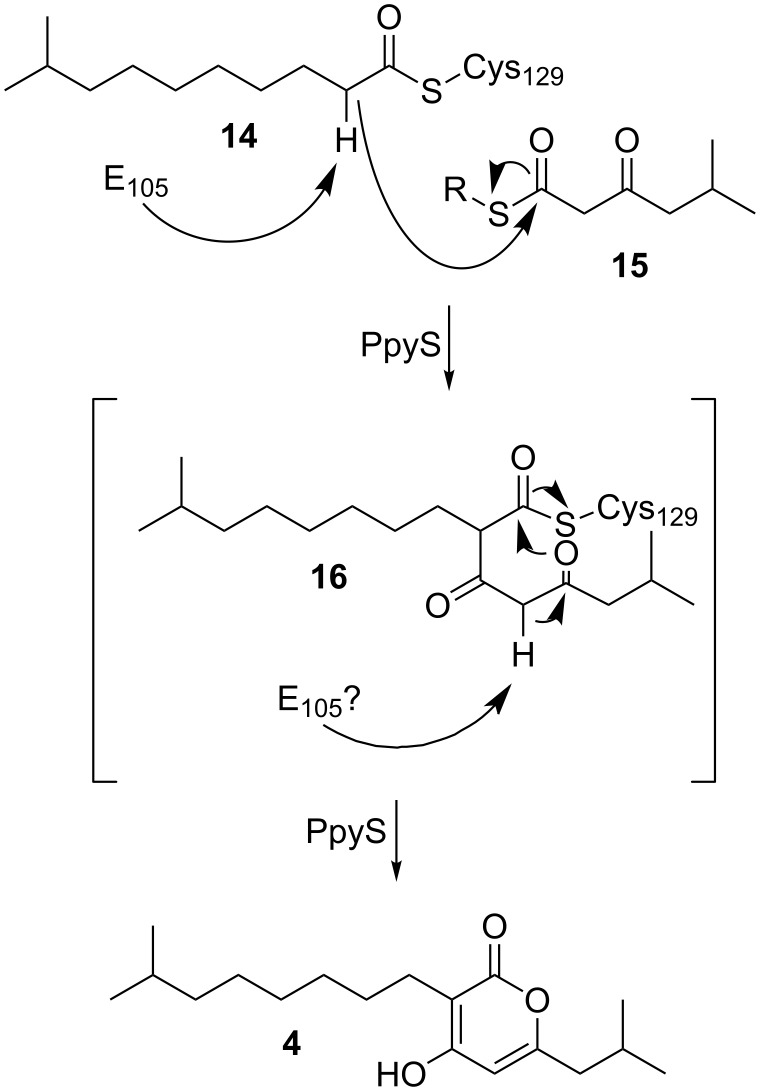
Proposed biosynthesis of photopyrone D (**4**) by PpyS from *P. luminescens.* The second deprotonation step can either occur spontaneously or is also catalyzed by E105.

Usually pyrones are derived in a one-chain mechanism from type III polyketide synthases [2,19] rarely showing two alkyl substituents resulting from the incorporation of different extender units [20]. Contrary to this, experiments using stable isotope labeled precursors [7] suggested a two-chain biosynthesis mechanism for photopyrone biosynthesis (Scheme 1): First, thioester-activated 9-methyldecanoic acid **14** is covalently bound to an active site cysteine. Deprotonation of the α-carbon of **14** results in the formation of a nucleophile, which subsequently attacks the carbonyl carbon of a 5-methyl-3-oxohexanoyl thioester **15**, formed by BkdABC [18] to form a new C–C bond. After an additional deprotonation of the bound intermediate **16** the α-pyrone ring is formed and **4** is released from PpyS. The two substrates **14** and **15** might be either ACP (acyl carrier protein) or CoA (coenzyme A) bound thioesters depending whether they originate from fatty acid biosynthesis or degradation, respectively. Due to the variability of the first substrate regarding chain length and starting unit, the different photopyrones A–H (**1–8**) are produced. We assume that in the second deprotonation step, the deprotonation can either occur spontaneously, due to the two neighboring carbonyl groups or it can be enzyme catalyzed.

To investigate the photopyrone biosynthesis in *P. luminescens*, we decided to generate a homology model for PpyS as no crystal structure could be obtained due to low expression levels under all conditions tested. The homodimeric structure of OleA from *Xanthomonas campestris*, showing the highest sequence identity (27%) of all available crystal structures at the PDB [21], was used as a template. OleA is responsible for the head-to-head condensation of two long-chain fatty acid thioesters, similar to the proposed mechanism of PpyS (Scheme 1). The dimeric structure of OleA revealed a unique feature that has not been described previously for other KS, in which one glutamic acid residue reaches inside the binding pocket of the adjacent monomer to catalyze the reaction by deprotonation of the enzyme-bound first acyl intermediate. For OleA, E117 of the β-monomer (E117β) reaches into the binding pocket of the α-monomer and vice versa.

**Figure 2:**
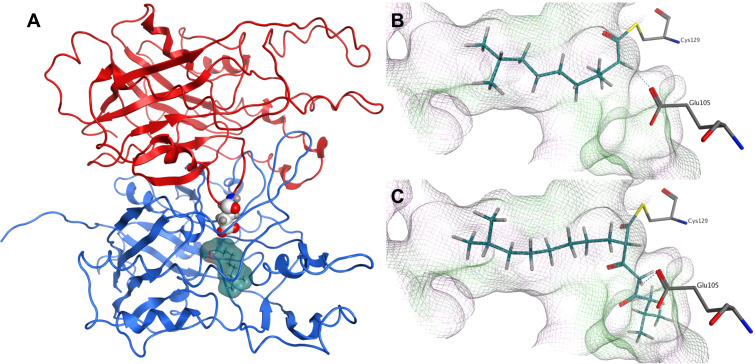
Dimeric structure of modeled PpyS (A). Chain A (blue), chain B (red). **14** (surface, cyan) is covalently docked to the active site at Cys129α and the atoms of Glu105β are represented as white spheres. Glu105 is proposed to act as a catalytic base. A detailed view of the proposed PpyS-binding pocket with covalently docked **14** (B) and **16** (C), respectively. The cavity of the binding pocket is shown in a line representation, where green represents a lipophilic, magenta a hydrophilic and white a neutral surface area. Possible hydrogen bonds are shown as dashed blue lines.

The generated homodimeric model of PpyS shows the catalytic triad (C129, H281, N310) responsible for the covalent binding of the fatty acid precursor, as well as amino acids present at the dimer interface (Figure S3, Supporting Information File 1). Using this model we performed docking studies by covalently docking **14** and **16** (Figure 2) into the active site C129, revealing that the binding cavity of modeled PpyS is capable of harboring both molecules. Similar to the OleA model, the PpyS model suggests glutamic acid E105 acting as a base by forming a hydrogen bond with the α-carbon of both covalently bound substrates. To confirm this hypothesis, a E105A derivative of PpyS was produced in *E. coli* resulting in the total loss of photopyrone production (Figures S1 and S2, Supporting Information File 1). Similarly, no photopyrone was produced in a R121D derivative. R121α is predicted by the model to be located at the dimer interface interacting with D137β. Thus, dimerization shown to be essential for KS activity [10–11] might be disturbed in a R121D variant resulting in a loss of KS activity.

Other examples of α-pyrones derived from a two-chain condensation of two acyl moieties are myxopyronin [22] and corallopyronin [23] that require a similar KS encoded by *mxnB* and *corB*, respectively. Both natural products have been shown to be potent antibiotics targeting the newly identified switch region of the bacterial RNA polymerase [24]. Furthermore the promiscuity of MxnB regarding its substrate specificity has been used in mutasynthesis experiments to produce novel myxopyronin derivatives [25]. Recently the crystal structure of MxnB has been solved [26]. Unlike PpyS, the proposed mechanism does not show a similar glutamic acid to be involved. A multiple sequence alignment of MxnB, CorB, PpyS and other KS (Figure S9, Supporting Information File 1) reveals that in both MxnB and CorB KS E105 in PpyS is replaced with V94. However, as the corresponding α-carbon is adjacent to two carbonyl groups (instead of one as in photopyrone biosynthesis) it might be deprotonated spontaneously due to the electron-withdrawing carbonyl functions.

**Figure 3:**
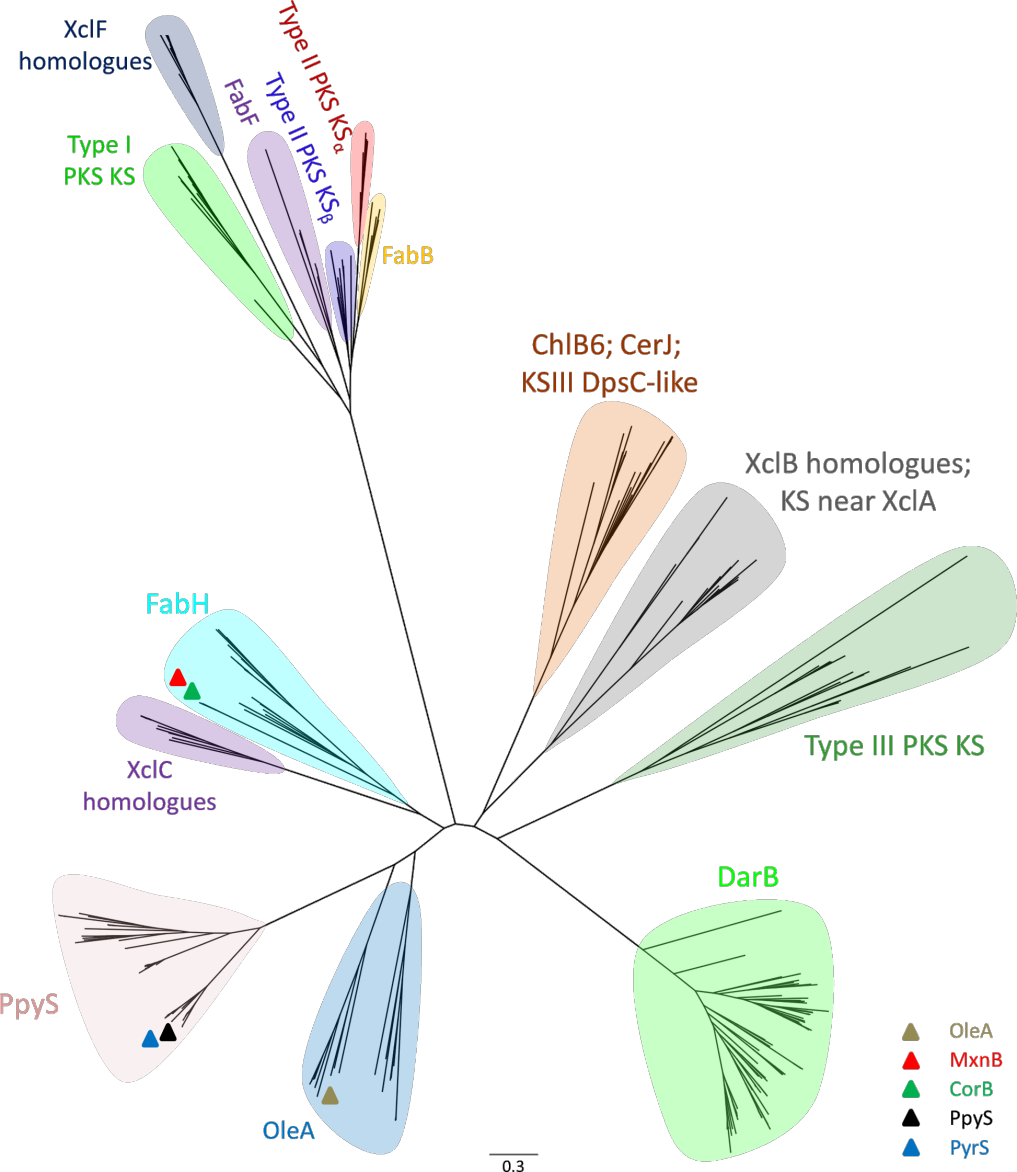
Phylogenetic tree (PHYML) composed of PpyS, its homologues and other known ketosynthases. Table S4 (Supporting Information File 1) lists all shown ketosynthases in this tree. The scale bar indicates the degree of divergence as substitutions per site.

To take a closer look into the evolutionary origin of PpyS, we compiled a collection of 284 different KS belonging to different families (Table S4, Supporting Information File 1) and reconstructed their phylogenetic tree (Figure 3). Using a BLASTP [27] search and the sequence of PpyS as query we included the top 72 hits into the collection. These sequences showed the highest similarity to PpyS and possess a glutamic acid at the same position as PpyS. Interestingly, OleA and PpyS and their homologues each form a novel KS branch and seem to be evolved from FabH-type KS. From the 72 identified PpyS homologues 21 appear to be more related to OleA then to PpyS as they cluster along with OleA. As expected, the remaining 51 sequences show a higher similarity to PpyS and the newly formed PpyS branch shows a separation into two distinct groups with PpyS and PyrS (pseudopyronine synthase) being present in one of them. The homologue PyrS from *Pseudomonas* sp. GM30 is described below. The biosynthetic importance of the second group including PpyS homologues from *Burkholderia*, *Legionella*, *Nocardia*, *Microcystis* and *Streptomyces* needs to be determined in future work. In contrary, MxnB and CorB are located within the FabH clade, showing that not only the reaction mechanism of these two KS is distinct from PpyS but also its evolutionary relationship, even if they produce a similar class of compound. Thus, the phylogenetic analysis is also useful to distinguish between reaction mechanisms of different KS.

In order to prove that the identified PpyS homologues are indeed involved in pyrone biosynthesis we analyzed the KS from *Pseudomonas* sp. GM30, as we knew that α-pyrone antibiotics pseudopyronine A (**9**) and B (**10**) have been isolated in two *Pseudomonas* strains but neither their biosynthesis nor the involved KS had been reported yet [28]. Both compounds have been described to show an antimycobacterial and antiparasitic activity and they inhibit the fatty acid biosynthesis [29]. Natural and unnatural derivatives have also been synthesized in order to find more potent compounds [30]. We first analyzed the strain for pseudopyronine production and were able to detect three possible compounds. After their isolation followed by NMR-based structure elucidation (Table S5, Supporting Information File 1) we could confirm that *Pseudomonas* sp. GM30 is producing pseudopyronine A (**9**), B (**10**) and the new derivative C (**11**) all differing in the chain length of the eastern acyl moiety. As heterologous expression of the proposed KS from strain GM30 in *E. coli* did not result in the production of **9**–**11** or a similar derivative, heterologous expression was tested in *Pseudomonas putida* KT2440 as well as homologous expression in *Pseudomonas* sp. GM30. In both experiments production of **9**–**11** was observed (Figure S7, Supporting Information File 1). Homologous expression in *Pseudomonas* sp. GM30 led to an increased production of all three compounds, confirming that the analyzed KS (‘gene name renamed PyrS for pseudopyronine synthase) is responsible for pseudopyronine biosynthesis. The homology model for PyrS (Figure S8, Supporting Information File 1) and docking experiments (Figure S9, Supporting Information File 1) with the proposed substrate **17** and intermediate **19** to the active site C124 also confirmed the glutamic acid inside the binding pocket as catalytically important suggesting a biosynthesis model (Scheme S1, Supporting Information File 1) similar to that of photopyrone (Scheme 1). Interestingly, PpyS and PyrS differ in substrate specificity for the acyl substrates. With this knowledge, it might now be possible to perform mutasynthesis experiments for the production of new, more potent compounds. It could also be possible that pseudopyronines might also serve as a communication signal in strain GM30 and other *Pseudomonas* strains, which are generally rich in cell–cell communication signals [31–35].

In summary we have shown that two unusual ketosynthases are responsible for the formation of photopyrones and pseudopyronines. Furthermore, we have confirmed the PpyS mechanism by mutagenesis and through detailed phylogenetic analysis we could show that PpyS and homologous KS do form a new branch in the KS phylogenetic tree that is more distant to other pyrone forming KS as found in the biosynthesis of the potent antibiotics myxopyronin and corallopyronin.

## Supporting Information

File 1 Experimental procedures, details of bioinformatic analysis and NMR data of pseudopyronines.
