# Supplementary material for: A novel and widespread class of ketosynthase is responsible for the head-to-head condensation of two acyl moieties in bacterial pyrone biosynthesis
Source: Beilstein J Org Chem. 2015 Aug 12;11:1412–7. doi: 10.3762/bjoc.11.152 (PMC4578411; doi:10.3762/bjoc.11.152)

Supporting Information

for

A novel and widespread class of ketosynthase is responsible for the head-to-head condensation of two acyl moieties in bacterial pyrone biosynthesis

Darko Kresovic[1], Florence Schempp[1], Zakaria Cheikh-Ali[1] and Helge B. Bode*[1,2]

Address: [1]Merck Stiftungsprofessur für Molekulare Biotechnologie, Fachbereich Biowissenschaften, Goethe Universität Frankfurt, 60438 Frankfurt am Main, Germany and [2]Buchmann Institute for Molecular Life Sciences (BMLS), Goethe Universität Frankfurt, 60438 Frankfurt am Main, Germany

Email: Helge B. Bode - h.bode@bio.uni-frankfurt.de
* Corresponding author

Experimental procedures, details of bioinformatic analysis and NMR data of pseudopyronines

Materials and methods

Cultivation of strains. *E. coli* strains were cultivated in liquid or solid LB-medium (10 g/L tryptone, 5 g/L yeast extract and 10 g/L NaCl) or TB-medium (12 g/L tryptone, 24 g/L yeast extract, 4 mL/L glycerol and 0.17 M KH_2PO_4/0.72 M K_2HPO_4). For preparation of solid media, 1.5% (w/v) Agar was added. For plasmid preparation *E. coli* strains were cultivated at 37 °C in TB-medium. Kanamycin (50 μg/mL) and chloramphenicol (20 μg/mL) were used as resistant markers. For cultivation of *Pseudomonas* sp. GM30 and *Pseudomonas putida* KT2440 the same media was used but strains were grown at 30 °C.

Construction of *ppyS* mutants. Mutants were either constructed using the TA-cloning strategy [1] or with oligonucleotide-directed mutagenesis. Point mutations were introduced to the pCOLA_*ppys* vector, which is used to heterologously express

PpyS in *E. coli* BL21(DE3) Star. Mutants C129A, H281A, E105A and E330A were constructed using the TA-cloning strategy, for this method a pair of oligonucleotides (v.p. fw and v.p. rev) were used to amplify the vector, containing the non-mutated gene section using the Phusion polymerase (Fermentas). After separation by agarose gel electrophoresis, the desired fragments were extracted with the GeneJET Gel Extraction Kit (Fermentas) and incubated for 30 minutes at 70 °C with Taq polymerase (Thermo Scientific), resulting in a 3' A-overhang fragment. Another pair of oligonucleotides (ds fw and ds rev), containing the mutated gene section, was used to form the 3' T-overhang fragment carrying the mutation. For ligation both fragments were incubated overnight at room temperature with T4 DNA ligase (Fermentas). The N310A and R121D PpyS mutants were created using oligonucleotide-directed mutagenesis, for which a pair of oligonucleotides was applied to amplify the vector and to introduce the point mutation using the Phusion polymerase (Fermentas) for PCR. For both strategies the ligation mixture was subsequently used to transform *E. coli* DH10B by electroporation (1250 V, 200 Ω and 25 μF). After plasmid extraction the obtained plasmids were verified by sequencing at SeqIT GmbH (Germany, Kaiserslautern). Verified plasmids were used to transform *E. coli* BL21 (DE3) Star along with pACYC_*bkdABC_ngrA* [2] by electroporation. For photopyrone biosynthesis both vectors (pCOLA_*ppyS* and pACYC_*bkdABC_ngrA*) are induced with 0.01 mM isopropyl-β-D-thiogalactopyranoside (IPTG) (Fermentas) for expression in *E. coli* BL21 (DE3) Star.

Cloning of pseudopyronine synthase (*pyrS*). We first constructed pCOLA_*pyrS* for heterologous expression in *E. coli* BL21 (DE3) Star. Therefore *pyrS* was cloned from extracted *Pseudomonas* sp. GM30 genomic DNA using the oligonucleotides *pyrS*_pCOLA_FW and *pyrS*_pCOLA_Rev. The vector pCOLADuet-1 and PyrS PCR

product were both digested with restriction enzymes BamHI and HindIII and ligated using the T4 DNA Ligase. This mixture was then used to transform *E. coli* DH10B by electroporation (1250 V). After plasmid extraction the obtained plasmid was verified by sequencing at SeqIT GmbH (Germany, Kaiserslautern). The verified plasmid was used to transform *E. coli* BL21 (DE3) Star by electroporation. The construction of pCom10_*pyrS* was performed using the Gibson assembly method [3]. Therefore the vector was amplified via PCR using the oligonucleotides pCom10_Fw and pCom10_Rev. PyrS was amplified using the oligonucleotides *pyrS*_pCom_Fw and *pyrS*_pCom_Rev and pCOLA_*pyrS* as template, the oligonucleotides were previously modified with a 30 bp 3' overhang which are homologues to the amplified pCom10 product. Both products were then incubated with the Gibson assembly mix for 1 h at 50 °C. This mixture was then used to transform *E. coli* DH10B by electroporation as described earlier. The plasmid was obtained by using the extraction protocol described previously.

Electrotransformation of *Pseudomonas* strains. *Pseudomonas putida* KT2440 and *Pseudomonas* sp. GM30 were grown over night at 30 °C in liquid LB media. To prepare cells for electro transformation 2 mL of fresh liquid LB media were inoculated with an overnight culture (1:100) and were then grown for 3 hours at 30 °C. The cells were then centrifuged and washed twice with cold water. Centrifuged cells were then resuspended in 50 μL cold water and cells were kept on ice. 1 μL of plasmid was used to transform *Pseudomonas* strains by electroporation (2500 V).

Analytical scale culture extraction. In order to detect photopyrone production in the wildtype and mutant strains by means of HPLC/MS, 20 mL of liquid LB-medium, containing the appropriate resistant markers, were inoculated with an overnight culture to an optical density of OD_{600} = 0.05 and cultivated for 3.5 h at 37 °C. Then

0.01 mM isopropyl-β-D-thiogalactopyranoside (IPTG) (Fermentas) and 2% Amberlite[TM] XAD16 (Sigma-Aldrich) were added to the culture, which was cultivated for 48 h at 16 °C. For detection of pseudopyronines 20 mL of liquid LB-medium, containing the appropriate resistant marker and 2% Amberlite[TM] XAD16, were inoculated with an overnight culture (1:100). Expression was induced with addition of 0.05% (v/v) dicyclopropyl ketone. The *Pseudomonas* containing cultures were then incubated for 72 h at 30 °C. After 48 h again 0.05% (v/v) of dicyclopropyl ketone was added. Cultures were harvested by centrifugation (4000 rpm, 10 min, 18 °C) followed by removal of the supernatant. Amberlite[TM] XAD16 resins were extracted with 30 mL of methanol and incubated for 1 h under constant rotation followed by a filtration step (Folded Filters (Quality), grade: 3 m/N, Munktell) to remove cells and resins. The elution step was repeated once with 10 mL of methanol. The methanol extract was then concentrated to dryness using a rotary evaporator. The solid residue was redissolved in 2 mL of methanol and a 1:10 dilution was analyzed by means of HPLC/MS. Extracts were analyzed using a Dionex UltiMate 3000 system coupled to a Bruker Daltonik AmaZon X mass spectrometer, a RP18-column (50 mm × 2.1 mm × 1.7 μm; Waters GmbH) and an acetonitrile/0.1% formic acid in H_2O gradient, ranging from 5 to 95% in 22 min at a flow rate of 0.6 mL/min. The production of *ppyS* mutants of **4** was calculated against standard concentrations of the main compound photopyrone D (**4**) produced by wildtype *ppyS*. The retention time of **4** under these conditions was 10.5 min.

Preparative extraction and purification. For the isolation of compounds **9–11** from *Pseudomonas* sp. GM30, the strain was cultivated in 6 L of LB-medium, with an addition of 2% Amberlite[TM] XAD16 for 3 days at 30 °C. Cultures were harvested by centrifugation (4000 rpm, 10 min, 18 °C) followed by removal of the supernatant.

Amberlite™ XAD16 resins were extracted with methanol and incubated for 1 h under constant rotation followed by a filtration step to remove cells and resins. The methanol extract was then concentrated to dryness using a rotary evaporator. The solid residue (4.6 g) was redissolved in 10 mL of water, then 20 mL of ethyl acetate was added and the mixture was shacked in a separating funnel. This step was repeated two times with the addition of 20 mL of ethyl acetate and separation. The ethyl acetate phase was then concentrated to dryness using a rotary evaporator. The solid residue (0.7 g) was redissolved in a 70% dimethyl sulfoxide (DMSO), 20% methanol and 10% isopropanol mixture. Compounds were then isolated with a Waters Bridge XBridgeTM Prep C18 5 μm OBD™ 19 × 150 mm Column (S/N) and a Waters HPLC-MS system as described in the following: Waters 3100 Mass Detector, Waters 2998 Photodiode Array Detector, Waters SFO System Fluidics Organizer, Waters 515 HPLC Pump, Waters 2545 Binary Gradient Module, Waters Selector Value, Waters 2767 Sample Manager. The purification was performed at a flow rate of 24 mL/min with an acetonitrile–water (0.1% formic acid) gradient: 0–26 min 50–75%, 26.1–30 min 76–95%. The combined fractions were then concentrated to dryness using a rotary evaporator to give 4.8 mg, 35.6 mg and 1.6 mg of **9**, **10** and **11**, respectively.

NMR. 1D and 2D nuclear magnetic resonance (NMR) spectra for purified compounds were recorded on a Bruker DRX 500 spectrometer using deuterated dimethyl sulfoxide as solvent and internal standard.

HR-ESI-MS. Determination of exact masses of **9**–**11** were carried out using a Dionex Ultimate 3000 RSLC coupled to a Bruker micrOTOF-Q II equipped with an ESI source. The following masses were detected: for **9,** *m/z* 267.1956 (*calcd* for $[C_{16}H_{26}O_3 + H]^+$, 267.1955, Δ = 0.38 ppm); **10**, *m/z* 295.2270 (*calcd* for $[C_{18}H_{30}O_3 +$

$H]^+$, 295.2268, Δ = 0.68 ppm); **11**, *m/z* 323.2583 (*calcd* for $[C_{20}H_{34}O_3 + H]^+$, 323.2581, Δ = 0.62 ppm).

Homology modelling. The protein sequences of PpyS and PyrS were used as queries for BLASTP [4] searches in the PDB [5], to identify the most similar available structure in the PDB. This resulted in the identification of OleA (sequence identity 27%, E-value 1e-14, PDB: 3S21) from *Xanthomonas campestris* for PpyS and OleA (sequence identity 37%, E-value 4e-10, PDB: 3S21) for PyrS-. These template structures were used to create a sequence alignment applying the ClustalW algorithm [6]. The homology models were generated using the Homology Modelling Tool integrated in MOE 2012.10 (Molecular Operating Environment; Chemical Computing Group Inc., Montreal, Canada) and the ClustalW sequence alignment was imported. A series of ten models was created, for further processing the one with the highest packing quality score was chosen and energy minimized applying the AMBER12EHT (integrated in MOE) force field. All figures showing protein structures in this work, were created using MOE.

Docking. Protein–ligand docking calculations were carried out using the program GOLD (version 5.2) [7] using the empirical scoring function for advanced protein–ligand docking CHEMPLP [8]. For each docking study the result with the highest docking score is shown in this work.

Phylogenetic analysis. A PHYML [9] tree (50 bootstraps) was calculated using a ClustalW alignment (gap opening: 10; gap extension: 0.1), which was generated using the collected ketosynthases. For visualization and calculation of the alignment as well as the PHYML tree the Geneious software (Biomatters Ltd., New Zealand) was used.

Supplementary Tables and Figures

Table S1: Strains used in this work.

Strain	Genotype	Reference
E. coli DH10B	F_ mcrA (*mrr-hsd*RMS-*mcr*BC), 80*lacZ* Δ, M15, Δ *lac*X74 *rec*A1 *end*A1 *ara*D 139 Δ (*ara, leu*)7697 *gal*U *gal*K λ *rpsL* (*Strr*) *nup*G	[10]
E. coli BL21 (DE3) Star	F- *omp*T *hsd*SB(rB-, mB-) *gal dcm rne*131 (DE3)	Invitrogen
Pseudomonas sp. GM30	Wildtype	[11]
Pseudomonas putida KT2440	Wildtype	[12]
WT	BL21 (DE3) Star:pCOLA_*ppyS*, pACYC_*bkd*ABC_*ngr*A, Km^R, Cm^R	[2]
C129A	BL21 (DE3) Star:pCOLA_*ppyS*_C129A, pACYC_*bkd*ABC_*ngr*A, Km^R, Cm^R	this work
H281A	BL21 (DE3) Star:pCOLA_*ppyS*_H281A, pACYC_*bkd*ABC_*ngr*A, Km^R, Cm^R	this work
N310A	BL21 (DE3) Star:pCOLA_*ppyS*_N310A, pACYC_*bkd*ABC_*ngr*A, Km^R, Cm^R	this work
E105A	BL21 (DE3) Star:pCOLA_*ppyS*_E105A, pACYC_*bkd*ABC_*ngr*A, Km^R, Cm^R	this work
R121D	BL21 (DE3) Star:pCOLA_*ppyS*_R121D, pACYC_*bkd*ABC_*ngr*A, Km^R, Cm^R	this work
E330A	BL21 (DE3) Star:pCOLA_*ppyS*_E330A, pACYC_*bkd*ABC_*ngr*A, Km^R, Cm^R	this work
E. coli DH10B pCOLA_*pyrS*	*E. coli* DH10B:pCOLA_*pyrS*	this work
E. coli BL21 (DE3) Star pCOLA_*pyrS*	*E. coli* BL21 (DE3) Star:pCOLA_*pyrS*	this work
E. coli DH10B pCom10_*pyrS*	*E. coli* DH10B:pCom10_*pyrS*	this work

P. putida KT2440 pCom10_*pyrS*	*Pseudomonas putida* KT2440:pCom10_*pyrS*	this work
Pseudomonas sp. Gm30 pCom10_*pyrS*	*Pseudomonas* sp. GM30:pCom10_*pyrS*	this work

Table S2: Plasmids used in this work.

Plasmid	Genotype	Reference
pCOLADuet-1	ColA ori, Km^R, T7lac promotor	Merck Millipore
pACYCDuet-1	CloDF13 ori, Cm^R, T7lac promotor	Merck Millipore
pCOLA_*ppyS*	ColA ori, Km^R, T7lac promotor, *ppyS*	[2]
pACYC_*bkd*ABC_*ngr*A	CloDF13 ori, Cm^R, T7lac promotor, *bkd*ABC, *ngr*A	[2]
pCOLA_*ppyS*_C129A	ColA ori, Km^R, T7lac promotor, *ppyS*	this work
pCOLA_*ppyS*_H281A	ColA ori, Km^R, T7lac promotor, *ppyS*	this work
pCOLA_*ppyS*_N310A	ColA ori, Km^R, T7lac promotor, *ppyS*	this work
pCOLA_*ppyS*_E105A	ColA ori, Km^R, T7lac promotor, *ppyS*	this work
pCOLA_*ppyS*_R121D	ColA ori, Km^R, T7lac promotor, *ppyS*	this work
pCOLA_*ppyS*_E330A	ColA ori, Km^R, T7lac promotor, *ppyS*	this work
pCom10	pBR322 ori, Km^R, *alkB* promotor	[13]
pCom10_*pyrS*	pBR322 ori, Km^R, *alkB* promotor, *pyrS*	this work
pCOLA_*pyrS*	ColA ori, Km^R, T7lac promotor, *pyrS*	this work

Table S3: Oligonucleotides used in this work.

Oligonucleotide	Sequence
Cys129_Fw.ds	CGCCGCGAACGGT
Cys129_Rev.ds	CCGTTCGCGGCGT
Cys129_Fw.V.P	TGGGTGGCAGCTATGGATATTATCAATAG
Cys129_Rev.V.P	CTACAACATCGTAGTTCCTACAAGTTAGCC
Glu105_Fw.ds	TAGCGCCAGCTAACAGTACTT
Glu105_Rev.ds	AGTACTGTTAGCTGGCGCTAT
Glu105_Fw.V.P	TTGTCTCAAAGGCACTAGGGCTAACTTGT
Glu105_Rev.V.P	GAAGCCGCGGGCAACACTGG
His281_Fw.ds	TTTTTATTGCGACAGGTTCACCAAAAACGT
His281_Rev.ds	CGTTTTTGGTGAACCTGTCGCAATAAAAAT
His281_Fw.V.P	GGGCACGCCTTGGTCAGAAGATG

His281_Rev.V.P	ATGTTCGATATCGTCAGATATTTTGTGATGATTAAA
Glu330_Fw.ds	TATTGGCGAAAAATCAACTTTGTATGGGATGGGCT
Glu330_Rev.ds	GCCCATCCCATACAAAGTTGATTTTTCGCCAATAT
Glu330_Fw.V.P	GGTAGTGGTGGAATGGTCTTTGCCGC
Glu330_Rev.V.P	ACCTTGATTAATGGCATCGAATATTCCGAACGG
Arg121Asp_Fw	GATAACTACGATGTTGTAGACGCCTGTAAC
Arg121Asp_Rev	ACAAGTTAGCCCTAGTGCCTTTGAGACAAA
Asn310_Fw	ATTGCTACAGCATCTATCCCGTTCGGAAT
Asn310_Rev	CGCACCTACCCGTTGGCCAAC
*pyrS*_pCOLA_FW	AGGATCCGATGAAAATAGTTGGGCTGTCC
*pyrS*_pCOLA_Rev	CGCAAGCTTTGCTACAAGGTAAACGACAGTGCA
pCom10_Fw	TCCAATTTTTATTAAATTAGTCGCTACGAG
pCom10_Rev	CTGTTTTGGCGGATGAGAGAAG
*pyrS*_pCom_Fw	CTCGTAGCGACTAATTTAATAAAAATTGGAATGAAAATAGTTGGGCTGTCC
*pyrS*_pCom_Rev	CTGAAAATCTTCTCTCATCCGCCAAAACAGCTACAAGGTAAACGACAGTGCAG

Table S4: Ketosynthases used for the phylogenetic tree. The sequences are ordered clockwise according to their location in the respective branches. All KS showing the conserved glutamic acid residue identified as catalytically important are shown in red.

	Protein	Organism	Accession number
	**OleA homologues**		
1	3-Oxoacyl-ACP synthase	*S.* sp. NRRL F-5555	WP_030402327
2	3-Oxoacyl-ACP synthase	*Streptomyces*	WP_031086294
3	3-Oxoacyl-ACP synthase	*S.* sp. NRRL F-5650	WP_031039494
4	3-Oxoacyl-ACP synthase	*A. rifamycini*	WP_026404743
5	3-Oxoacyl-ACP synthase	*M. rosea*	WP_036407026
6	3-Oxoacyl-ACP synthase	*N. candida*	WP_043622914
7	3-Oxoacyl-ACP synthase	*S. pristinaespiralis*	WP_005309093
8	3-Oxoacyl-ACP synthase	*T.* sp. 28	WP_045191732
9	3-Oxoacyl-ACP synthase	*S. hofmanni*	WP_017748751
10	3-Oxoacyl-ACP synthase	*L. araneosa*	WP_007281261
11	OleA	*X. campestris* pv. *campestris*	3S21_A
12	3-Oxoacyl-ACP synthase	*S. amylolyticus*	AKF07269
13	3-Oxoacyl-ACP synthase	*B. muris*	WP_017822397
14	3-Oxoacyl-ACP synthase	*A. phenanthrenivorans*	WP_043453199
15	3-Oxoacyl-ACP synthase	*Arthrobacter*	WP_018779660
16	3-Oxoacyl-ACP synthase	*A.* sp. MWB30	KIA73109
17	3-Oxoacyl-ACP synthase	*L. rubra*	WP_021808728
18	3-Oxoacyl-ACP synthase	*M. yannicii*	WP_040569064
19	3-Oxoacyl-ACP synthase	*M.* sp. B19	WP_026096098
20	3-Oxoacyl-ACP synthase	*M. testaceum*	WP_043360932
21	3-Oxoacyl-ACP synthase	*M.* testaceum StLB037	BAJ73499
22	3-Oxoacyl-ACP synthase	*M.* sp. SUBG005	KEP74827
	**PpyS homologues**		
23	3-Oxoacyl-ACP synthase	*X. nematophila*	WP_010847197
24	3-Oxoacyl-ACP synthase	*X. nematophila* ATCC 19061	YP_003713506

25	3-Oxoacyl-ACP synthase	*X. nematophila*	WP_013184973
26	3-Oxoacyl-ACP synthase	*X. bovienii*	WP_038246124
27	3-Oxoacyl-ACP synthase	*X. bovienii*	WP_038180969
28	PpyS	*P. luminescens* TT01	AGO97060
29	3-Oxoacyl-ACP synthase	*P. luminescens* TT01	CAE17216
30	3-Oxoacyl-ACP synthase	*P. luminescens* TT01	WP_046396397
31	3-Oxoacyl-ACP synthase	*P.* sp. PH1b	WP_025131331
32	3-Oxoacyl-ACP synthase	*P.* sp. St29	BAQ80432
33	3-Oxoacyl-ACP synthase	*P.* sp. Os17	BAQ74133
34	3-Oxoacyl-ACP synthase	*P. mosselii*	WP_023630065
35	3-Oxoacyl-ACP synthase	*P. mosselii*	WP_028692573
36	PyrS	*P.* sp. GM30	WP_007967127
37	3-Oxoacyl-ACP synthase	*P.* sp. URIL14HWK12:I6	WP_027611766
38	3-Oxoacyl-ACP synthase	*P.* sp. W15Feb9B	WP_041064001
39	3-Oxoacyl-ACP synthase	*B.* sp. UYPR1.413	WP_028370407
40	3-Oxoacyl-ACP synthase	*B. bannensis*	WP_027819887
41	3-Oxoacyl-ACP synthase	*B. mimosarum*	WP_028232503
42	3-Oxoacyl-ACP synthase	*B. nodosa*	WP_028204568
43	3-Oxoacyl-ACP synthase	*B. heleia*	WP_042262981
44	3-Oxoacyl-ACP synthase	*B. phytofirmans* PsJN	YP_001889944
45	3-Oxoacyl-ACP synthase	*B. phytofirmans*	WP_012428081
46	3-Oxoacyl-ACP synthase	*B.* sp. WSM2230	WP_025596424
47	3-Oxoacyl-ACP synthase	*B.* sp. WSM2232	WP_027214818
48	3-Oxoacyl-ACP synthase	*B.* sp. CCGE1003	YP_003910175
49	3-Oxoacyl-ACP synthase	*B.* sp. CCGE1003	WP_013342411
50	3-Oxoacyl-ACP synthase	*B.* sp. WSM3556	WP_027802201
51	3-Oxoacyl-ACP synthase	*B. graminis*	WP_006049675
52	3-Oxoacyl-ACP synthase	*B.* sp. URHA0054	WP_029966890
53	3-Oxoacyl-ACP synthase	*B.* sp. CCGE1001	YP_004230959
54	3-Oxoacyl-ACP synthase	*B.* sp. CCGE1001	WP_013591004
55	3-Oxoacyl-ACP synthase	*B. phenoliruptrix* BR3459a	YP_006793509
56	3-Oxoacyl-ACP synthase	*Burkholderia*	WP_014972441
57	3-Oxoacyl-ACP synthase	*L. anisa*	WP_019234674
58	3-Oxoacyl-ACP synthase	*L. pneumophila*	WP_028378564
59	3-Oxoacyl-ACP synthase	*S.* sp. CNB091	WP_018955464
60	3-Oxoacyl-ACP synthase	*A. mirum*	WP_015801934
61	3-Oxoacyl-ACP synthase	*A. azurea*	WP_005164847
62	3-Oxoacyl-ACP synthase	*S.* sp. MspMP-M5	WP_018537413
63	3-Oxoacyl-ACP synthase	*N. abscessus*	WP_043690931
64	3-Oxoacyl-ACP synthase	*N.* sp. CNY236	WP_028478668
65	3-Oxoacyl-ACP synthase	*N. farcinica*	WP_011207969
66	3-Oxoacyl-ACP synthase	*N. higoensis*	WP_040795117
67	3-Oxoacyl-ACP synthase	*G. mallensis*	WP_014267925
68	3-Oxoacyl-ACP synthase	*T. campylonemoides*	WP_041033411
69	3-Oxoacyl-ACP synthase	*A.* sp. PCC 7108	WP_016949109
70	3-Oxoacyl-ACP synthase	*M.* sp. SC2	WP_014890909
71	3-Oxoacyl-ACP synthase	*M. rosea*	WP_018405831
72	3-Oxoacyl-ACP synthase	*M.* sp. SB2	WP_029650722
73	3-Oxoacyl-ACP synthase	*M.* sp. T1-4	WP_008198039
74	3-Oxoacyl-ACP synthase	*C. fritschii*	WP_016876568
	**Closest BLAST-P hits for XclC [14]**		
75	3-Oxoacyl-ACP synthase	*C. acetobutylicum*	NP_347450.1
76	3-Oxoacyl-ACP synthase	*P. lactis*	WP_007130623.1
77	3-Oxoacyl-ACP synthase	*B. thuringiensis*	YP_006930640.1
78	3-Oxoacyl-ACP synthase	*B.* sp. 1NLA3E	YP_007911827.1
79	3-Oxoacyl-ACP synthase	*O. scapharcae*	WP_010098042.1
80	3-Oxoacyl-ACP synthase	*P. polymyxa*	YP_003947618.1
81	3-Oxoacyl-ACP synthase	*P. polymyxa*	YP_003871436.1
82	3-Oxoacyl-ACP synthase	*P.* sp. Aloe-11	WP_007431139.1
83	3-Oxoacyl-ACP synthase	*P. terrae*	YP_005077926.1

84	3-Oxoacyl-ACP synthase	*P. peoriae*	WP_010345468.1
	**FabH**		
85	CorB	*C. coralloides*	ADI59524
86	Myxopyronin ketosynthase	*M. fulvus*	AGS77282
87	FabHB	*B. subtilis*	NP_388898
88	FabH	*N. punctiforme*	YP_001865657
89	3-oxoacyl-ACP synthase	*B. subtilis*	NP_389015.1
90	FabH	*A. fabrum*	NP_354198
91	FabH	*P. luminescens*	NP_930069
92	FabH	*E. coli*	NP_287225
93	FabH	*S. griseus*	YP_001826619
94	FabH	*S. echinatus*	AAV84077
95	NP_626634	*S. coelicolor* A3(2)	NP_626634
96	FabH	*S. avermitilis*	BAC73499
97	Q54206	*S. glaucescens*	Q54206
98	FdmS	*S. griseus*	AAQ08929
99	CAM58805_S._sp._BenQ	*S.* sp. A2991200	CAM58805
100	ZhuH 1MZJ	*S.* sp. R1128	AAG30195
101	FrnI	*S. roseofulvus*	AAC18104
102	AlnI	*S.* sp CM020	ACI88883
	**KS type I PKS**		
103	Plu1885	*P. luminescens*	NP_929153
104	NanA8	*S. nanchangensis*	AAP42874
105	EryAII	*S. erythraea*	YP_001102990
106	TylGI KSQ	*S. fradiae*	AAB66504
107	MerA	*S. violaceusniger*	ABJ97437
108	TamAI	*S.* sp. 3079	ADC79637
109	OleAI KSQ	*S. antibioticus*	AAF82408
110	HedT	*S. griseoruber*	AAP85336
	**Closest BLAST-P hits for XclF**		
111	3-Oxoacyl-ACP synthase	*R. blandensis*	WP_008043745.1
112	3-Oxoacyl-ACP synthase	*X. nematophila*	YP_003714026.1
113	3-Oxoacyl-ACP synthase	*X. nematophila*	WP_010848687.1
114	3-Oxoacyl-ACP synthase	*M.* sp. PE36	WP_006034384.1
115	3-Oxoacyl-ACP synthase	*P. profundum*	YP_132684.1
116	3-Oxoacyl-ACP synthase	*P. damselae*	WP_005305524.1
117	3-Oxoacyl-ACP synthase	*P.* sp. AK15	WP_007465048.1
118	3-Oxoacyl-ACP synthase	*P. leiognathi*	WP_008989540.1
119	3-Oxoacyl-ACP synthase	*P.* sp. SKA34	WP_006644045.1
120	3-Oxoacyl-ACP synthase	*P. angustum*	WP_005364526.1
	**FabF**		
121	FabF	*M.* sp. 4-46	YP_001771620
122	FabF	*C. pinensis*	ACU62401
123	cpin1855	*C. pinensis*	YP_003121552
124	Dfer_1997	*D. fermentans*	YP_003086385
125	FabB	*A. pleuropneumoniae*	ZP_00134992
126	FabB	*C.* sp. 30_2	ZP_04562837
127	NP_416826	*E. coli*	NP_416826
128	FabB	*S. boydii*	YP_001881145
	**Type II PKS KS b**		
129	NP_344945	*S. pneumoniae*	NP_344945
130	FabF	*T. thermophilus*	YP_143679
131	FabF	*N. punctiforme*	YP_001867862
132	FabF	*B. subtilis*	NP_389016
133	NP_645683	*S. aureus*	NP_645683
134	FabF	*P. luminescens*	NP_930065
135	FabF	*E. albertii*	ZP_02902779.1
136	FabF	*E. coli*	NP_287229
137	NP_415613	*E. coli*	NP_415613
138	FabF	*S. avermitilis*	BAC70003
	**Type II PKS KS a**		

139	SimA2	*S. antibioticus*	AF324838_4
140	TcmL	*S. glaucescens*	AAA67516
141	EncB	*S. maritimus*	AAF81729
142	ActIA	*S. coelicolor* A3(2)	SCO5087
143	NcnB	*S. arenae*	AAD20268
	**FabB**		
144	AntD (Plu4191)	*P. luminescens*	NP_931374
145	EncA	*S. maritimus*	AAF81728
146	ActiB	*S. coelicolor* A3(2)	SCO5088
147	NcnA	*S. arenae*	AAD20267
148	TcmK	*S. davawensis*	CCK26894
149	SimA1	*S. antibioticus*	AAK06784
	**ChlB6; CerJ; KSIII DpsC-like**		
150	ChlB6	*S. antibioticus*	AAZ77679
151	CerJ	*S. tendae*	AEI91069
152	CosE	*S. olindensis*	ABC00733
153	DpsC	*S. peucetius*	AAA65208
154	AknE2	*S.* sp. SPB74	ZP_04991255.1
155	AknE2	*S. galilaeus*	AAF70109
156	BAB72048	*S. galilaeus*	BAB72048
157	PokM2	*S. diastatochromogenes*	ACN64832
158	CalO4	*S. aurantiaca*	ZP_01462124
159	FabH	*S. erythraea*	YP_001107471
160	NdasDRAFT_3133	*N. dassonvillei*	ZP_04334033.1
161	ChlB3	*S. antibioticus*	AAZ77676
162	CalO4	*M. echinospora*	AAM70354
163	AviN	*S. viridochromogenes*	AAK83178
164	PlaP2	*S.* sp. Tu6071	ABB69750
165	CouN2	*S. rishiriensis*	AAG29787
166	CloN2	*S. roseochromogenes*	AAN65231
	**Closest BLAST-P hits for XclB**		
167	3-Oxoacyl-ACP synthase III	*B.* sp. EniD312	WP_009111263.1
168	3-Oxoacyl-ACP synthase III	*A. nasoniae*	CBA73264.1
169	3-Oxoacyl-ACP synthase III	*P. carotovorum*	WP_010301235.1
170	3-Oxoacyl-ACP synthase III	*P. pacifica*	WP_006975318.1
171	3-Oxoacyl-ACP synthase III	*C. stagnale*	YP_007317906.1
172	3-Oxoacyl-ACP synthase III	*N. punctiforme*	YP_001865628.1
173	3-Oxoacyl-ACP synthase III	*R.* sp. PCC 7116	YP_007056099
174	3-Oxoacyl-ACP synthase III	*S. cyanosphaera*	YP_007130807.1
175	3-Oxoacyl-ACP synthase III	*Calothrix* sp. PCC 6303	YP_007138278
176	3-Oxoacyl-ACP synthase III	*N. punctiforme*	YP_001868566.1
177	3-Oxoacyl-ACP synthase III	*R.*sp. PCC 7116	YP_007057764.1
	**KS adjacent to XclA homologues**		
178	3-Oxoacyl-ACP synthase	*C.* sp. PCC 7822	YP_003899922.1
179	3-Oxoacyl-ACP synthase	*N. punctiforme*	YP_001865657.1
180	3-Oxoacyl-ACP synthase	*A. cylindrica*	YP_007155727.1
	**KS type III PKS**		
181	Chs-like	*R. baltica*	NP_868579
182	BPS (PLN03172)	*H. androsaemum*	Q8SAS8
183	CHS H. (PLN03173)	*H. androsaemum*	Q9FUB7
184	CHS9	*M. sativa*	AAA02827
185	STS	*P. quinquefolia*	AAM21773
186	BAS	*R. palmatum*	AAK82824
187	bpsA	*B. subtilis* str. 168	NP_390087
188	MXAN_6639	*M. xanthus*	YP_634756
189	PKS10	*M. tuberculosis*	NP_216176
190	PKS11	*M. tuberculosis*	NP_216181
191	Cpz6 Capramyzin ketosynthase	*Streptomyces* sp. MK730–62F2	
192	Germicidin synthase	*Streptomyces coelicolor*	3V7I_A
193	RppA S	*S. antibioticus*	BAB91443

194	RppA	*S. avermitilis*	NP_828307
195	RppB	*S. antibioticus*	BAB91444
	**DarB**		
196	O3I_37171	*N. brasiliensis*	ZP_09843377
197	M446_0174	*M*. sp. 4-46	YP_001767187
198	cpin6850	*C. pinensis*	YP_003126452
199	BFO_3187	*T. forsythia*	YP_005015826
200	NiasoDRAFT_0547	*N. soli*	ZP_09632794
201	Mucpa_6793	*M. paludis*	ZP_09618305
202	Oweho_0889	*O. hongkongensis*	YP_004988545
203	CHU_0390	*C. hutchinsonii*	YP_677020
204	Fluta_1447	*F. taffensis*	YP_004344279
205	Dfer_5797	*D. fermentans*	YP_003090150
206	BZARG_2045	*B. argentinensis*	ZP_08820341
207	Lacal_2074	*L*. sp. 5H-3-7-4	YP_004580348
208	Aeqsu_0932	*A. sublithincola*	YP_006417450
209	Zobellia_2074	*Z. galactanivorans*	YP_004736513
210	Lbys_1508	*L. byssophila*	YP_003997574
211	HMPREF0204_10987	*C. gleum*	ZP_07085127
212	PMI13_02465	*C*. sp. CF314	ZP_10726507
213	HMPREF0156_01383	*B*. taxon 274 str. F0058	ZP_06983320
214	HMPREF9071_0527	*C*. taxon 338 str. F0234	ZP_08201061
215	CAPGI0001_0843	*C. gingivalis*	ZP_04056582
216	HMPREF1154_2288	*C*. sp. CM59	ZP_10880679
217	HMPREF1320_1701	*C*. taxon 335 str. F0486	EJF37460
218	HMPREF1321_1154	*C*. taxon 412 str. F0487	ZP_10366882
219	CAPSP0001_1216	*C. sputigena*	ZP_03390203
220	Coch_0547	*C. ochracea*	YP_003140666
221	HMPREF1319_0374	*C. ochracea*	EJF43732
222	HMPREF1977_1456	*C. ochracea*	ZP_07866642
223	Weevi_1554	*W. virosa*	YP_004238832.1
224	HMPREF9716_01579	*M. odoratimimus*	EKB07937
225	Myrod_1723	*M. odoratus*	ZP_09672239
226	HMPREF9711_01694	*M. odoratimimus*	EKB04829
227	HMPREF9712_01161	*M. odoratimimus*	ZP_09523568
228	Fcol_11845	*F. columnare*	YP_004942963
229	FP2279	*F. psychrophilum*	YP_001297136
230	PMI10_02641	*F*. sp. CF136	ZP_10730768
231	FF52_12311	*F*. sp. F52	ZP_10481912
232	Fjoh_1102	*F. johnsoniae*	YP_001193454
233	FJSC11DRAFT_3961	*F*. sp. JSC-11	ZP_08987753
234	MICAG_1820011	*M. aeruginosa*	CCI22605
235	DP1817	*D. psychrophila*	YP_065553
236	DaAHT2_1139	*D. alkaliphilus*	YP_003690456
237	MldDRAFT_4065	delta proteobacterium MLMS-1	ZP_01289639
238	CBGD1_514	*S. gotlandica*	ZP_05070248
239	SMGD1_1386	*S. gotlandica*	EHP29910
240	Sdel_2118	*S. deleyianum*	YP_003305165
241	Sulba_2257	*S. barnesii*	YP_006405107
242	Arnit_2310	*A. nitrofigilis*	YP_003656468
243	HMPREF9401_0244	*A. butzleri*	ZP_07890833
244	Hbal_2902	*H. baltica*	YP_003061270
245	ParcA3_010100003428	*P. arctica*	ZP_10280196
246	PspoU_010100018642	*P. spongiae*	ZP_10300425
247	PSJM300_17945	*P. stutzeri*	AFN79642
248	MDS_0597	*P. mendocina*	YP_004378380
249	Psefu_0435	*P. fulva*	YP_004472512
250	Plu2164	*P. luminescens*	NP_929424
251	PA-RVA6-3077	*P. asymbiotica*	CAR66906
252	PAU_02401	*P. asymbiotica*	YP_003041237
253	PchlO6_4243	*P. chlororaphis*	ZP_10172862

254	DarB	*P. chlororaphis*	AAN18032
255	Pchl3084_3967	*P. chlororaphis*	EJL05977
256	PMI20_00702	*P*. sp. GM17	ZP_10707840
257	Daro_2368	*D. aromatica*	YP_285574
258	azo0292 DarB	*A*. sp. BH72	YP_931796
259	Rfer_3974	*R. ferrireducens*	YP_525203
260	Slit_0359	*S. lithotrophicus*	YP_003522988
261	PMI12_02025	*V*. sp. CF313	ZP_10567997
262	Vapar_3389	*V. paradoxus*	YP_002945272
263	Varpa_2231	*V. paradoxus*	YP_004154548
264	COI_2002	*M. haemolytica*	ZP_05992665
265	COK_0379	*M. haemolytica*	ZP_05988513
266	HMPREF9417_0595	*H. parainfluenzae*	ZP_08147854
267	HMPREF9952_1824	*H. pittmaniae*	ZP_08755481
268	HMPREF9064_0174	*A.segnis*	ZP_07888807
269	ATCC33389_0196	*A. aphrophilus*	EGY32238
270	NT05HA_1737	*A. aphrophilus*	YP_003008155
271	HMPREF9335_01583	*A. aphrophilus*	EHB89432
272	GCWU000324_02596	*K. oralis*	ZP_04603113
273	EIKCOROL_00456	*E. corrodens*	ZP_03712789
274	HMPREF9371_1043	*N. shayeganii*	ZP_08886538
275	HMPREF9370_1914	*N. wadsworthii*	ZP_08940206
276	NEIFLAOT_02523	*N. flavescens*	ZP_03720660
277	HMPREF0604_01363	*N. mucosa*	ZP_07993739
278	NEIFL0001_0036	*N. flavescens*	ZP_04757628
279	NEISUBOT_03200	*N. subflava*	ZP_05983976
280	NEISICOT_02133	*N. sicca*	ZP_05318975
281	HMPREF9418_1128	*N. macacae*	ZP_08684521
282	HMPREF1051_1749	*N. sicca*	EIG27057
283	HMPREF1028_00835	*N*. sp. GT4A_CT1	ZP_08888860
284	HMPREF9016_01947	*N*. taxon 014 str. F0314	ZP_06980826

Table S5: NMR spectroscopic data (400 MHz, *J* in Hz) of pseudopyronine A, B and C in DMSO-d_6.

	Pseudopyronine A		Pseudopyronine B		Pseudopyronine C	
**Position**	**δ_C**	**δ_H *(J in Hz)***	**δ_C**	**δ_H *(J in Hz)***	δ_C	**δ_H *(J in Hz)***
**2**	164.9	-	165.0	-	166.9	-
**3**	100.7	-	101.3	-	101.5	-
**3a**	22.7	2.23 (t, 8.0, 2H)	22.7	2.24 (t, 8.0, 2H)	22.8	2.22 (t, 8.0, 2H)
**3b**	27.5	1.34 (m, 2H)	27.5	1.35 (t br, 8.0, 2 H)	27.7	1.34 (t br, 8.0, 2 H)
**3c**	28.5	1.24 (m, 2H)	28.6	1.26, (m, 2H)	28.8	1.23, (m, 2H)
**3d**	30.4	1.28 (m, 2H)	31.2	1.25 (m, 2H)	31.2	1.24 (m, 2H)
**3e**	22.0	1.24 (m, 2H)	22.0	1.26 (m, 2H)	22.1	1.25 (m, 2H)
**3f**	13.8	0.86 (m, 3H)	14.0	0.84 (m, 3H)	13.9	0.85 (m, 3H)
**4**	162.2	-	164.8	-	165.1	-
**5**	99.9	5.91 (s, 1H)	99.2	5.94 (s, 1H)	100.4	5.86 (s, 1H)
**6**	162.2	-	162.6	-	162.0	-
**6a**	32.5	2.36 (t, 8.0, 2H)	32.6	2.37 (t, 8.0, 2H)	32.6	2.34 (t, 8.0, 2H)
**6b**	25.8	1.52 (m, 2H)	26.2	1.51 (t br, 8.0, 2H)	26.2	1.50 (t br, 8.0, 2H)
**6c**	31.1	1.26 (m, 2H)	28.6	1.26 (m, 2H)	28.6	1.26 (m, 2H)
**6d**	21.7	1.26 (m, 2H)	28.2	1.28 (m, 2H)	28.2	1.26 (m, 2H)
**6e**	13.7	0.86 (m, 3H)	31.1	1.25 (m, 2H)	28.6	1.28 (m, 2H)
**6f**			22.0	1.26 (m, 2H)	28.6	1.28 (m, 2H)
**6g**			13.8	0.84 (m, 3H)	31.2	1.26 (m, 2H)
**6h**					22.1	1.26 (m, 2H)
**6i**					13.9	0.85 (m, 3H)

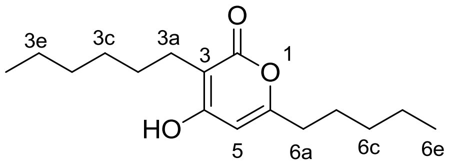

Pseudopyronine A

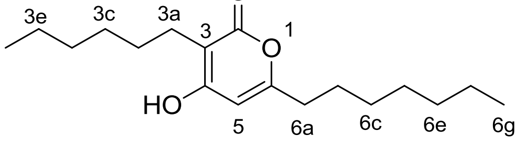

Pseudopyronine B

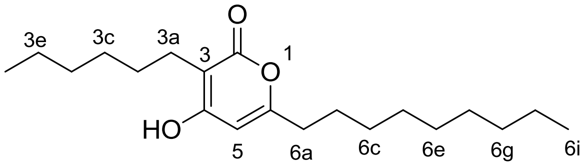

Pseudopyronine C

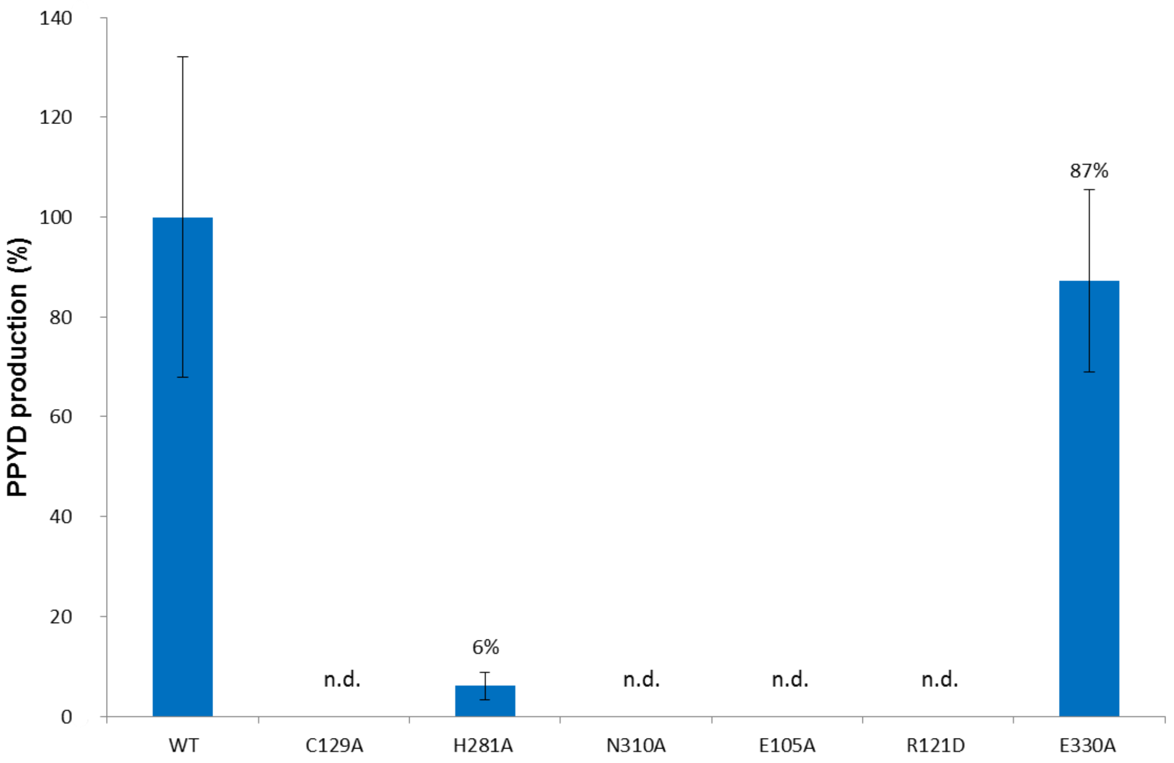

Figure S1: Analysis of amino acid substitutions for PpyS activity regarding the biosynthesis of photopyrone D (PPYD, **4**). The data is calculated in dependence to the production of the PpyS wildtype enzyme. Cys129, His281 and Asn310 form the catalytic triade, Glu105 is proposed to act as a catalytic base, Arg121 is located at the dimerization interface and Glu330 was used as a neutral control. In PpyS-C129A, -N310A, -E105A and -R121D the production of **4** was not detectable (n. d.) anymore.

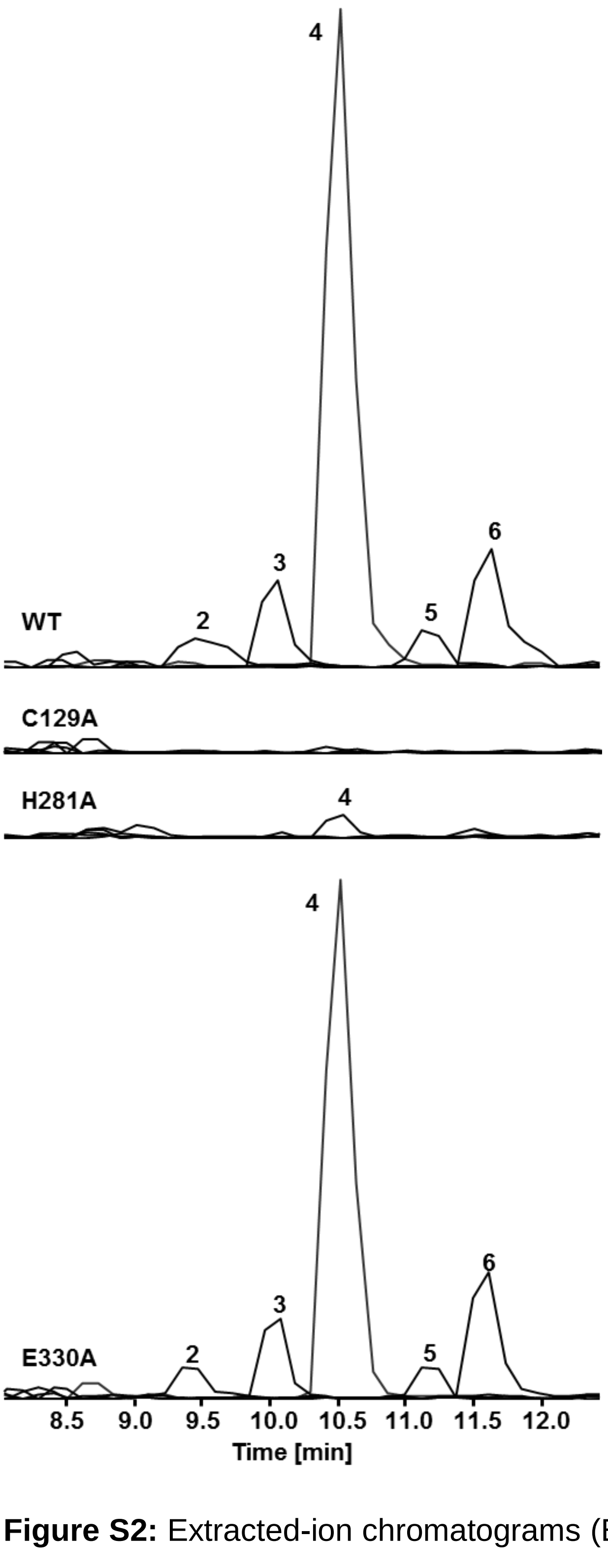

Figure S2: Extracted-ion chromatograms (EICs) of the most abundant photopyrones, which are produced heterologously in *E. coli* wildtype and mutant strains. **2** (*m*/*z* 267.2 $[M + H]^+$), **3** (*m*/*z* 281.2 $[M + H]^+$), **4** (*m*/*z* 295.2 $[M + H]^+$), **5** (*m*/*z* 309.2 $[M + H]^+$), **6** *m*/*z* 323.3 $[M + H]^+$). As the data for PpyS N310A, E105A and R121D look identical to C129A showing a complete loss of photopyrone production, they are not shown here.

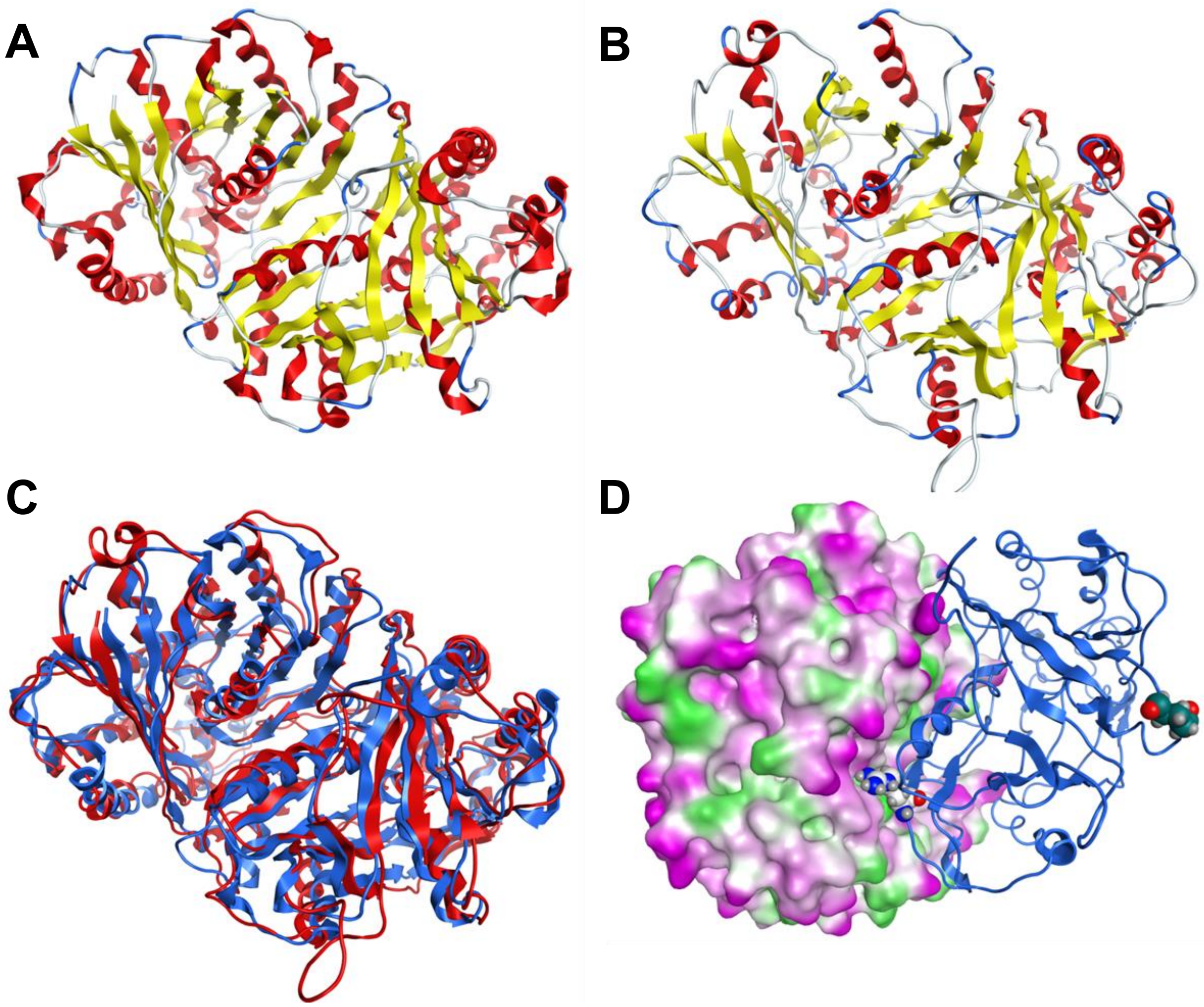

Figure S3: Structure of OleA-dimer (PDB: 3ROW) from *Xanthomonas campestris* (A). Modeled structure of PpyS-dimer from *P. luminescens* TT01 (B), which was generated using the OleA structure as a template: Red (α-helices), yellow (β-sheets), blue (turns), white (random coils). The superposition of OleA (blue) and PpyS (red) structures (C) revealed a root-mean-square deviation (RMSD) of 2.0 Å. To picture the dimer interface of PpyS the surface of chain α was calculated (green, magenta, and white represent a lipophilic, hydrophilic and neutral surface area, respectively) and the ribbon of chain β is shown (D). At the interface the atoms of Arg121β are represented as spheres, this residue is predicted to be involved in the dimerization of PpyS by interacting with Asp137α. The mutation of Arg121 led to a complete loss of photopyrone production. Furthermore Glu330 is shown in cyan spheres, this residue was mutated as a control, which should not influence the photopyrone production as indeed shown in Figure S1.

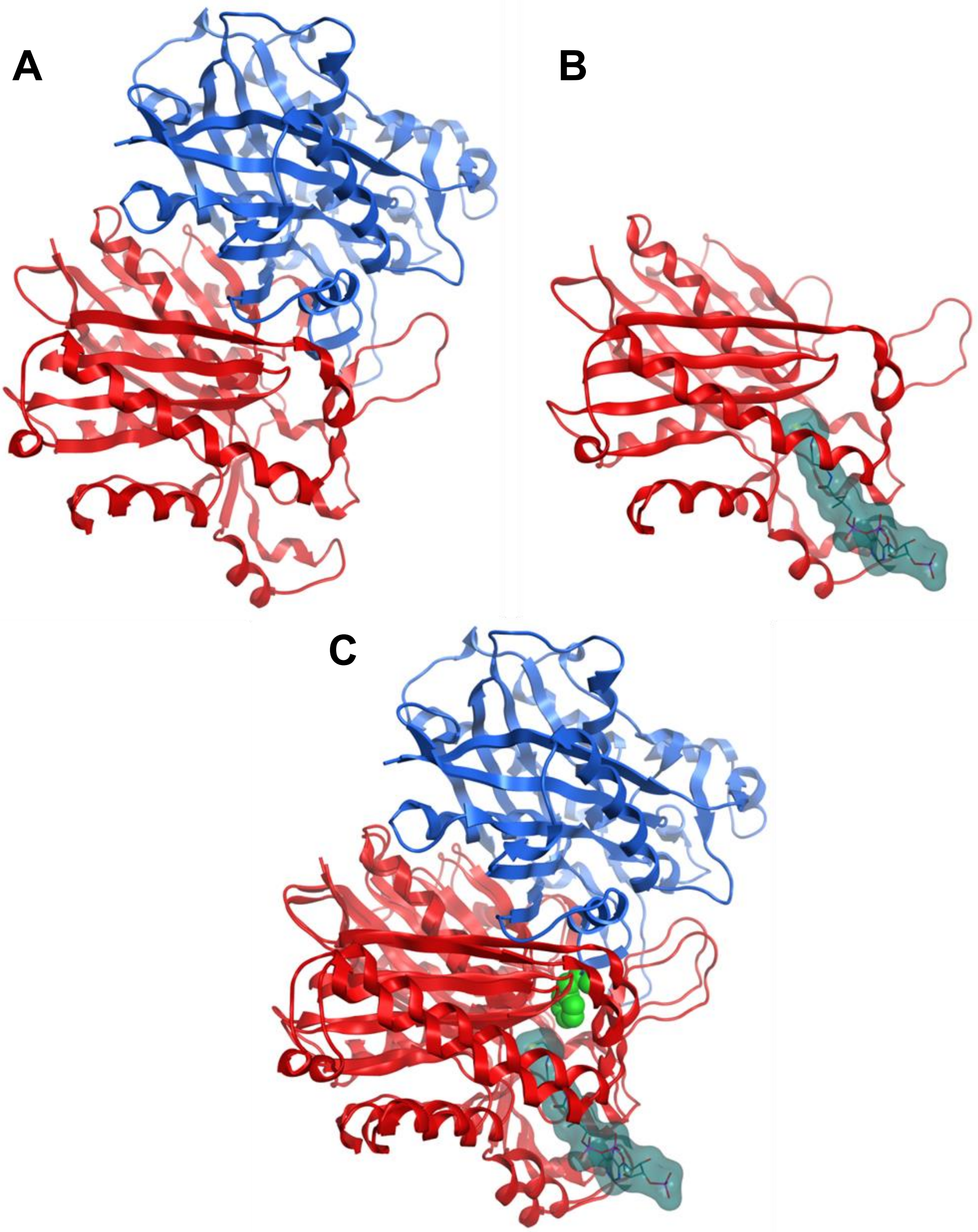

Figure S4: Structure of the FabH-dimer (PDB: 1HN9) from *E. coli* (A). Structure of FabH-monomer (PDB: 1HNJ) from *E. coli* along with co-crystallized malonyl-CoA (B). The superposition of both these structures (C) reveals the location of the substrate malonyl-CoA within the dimeric structure and the distance to Phe87β, which is the analogue to Glu105 of PpyS. With a shortest distance of ca. 5.3 Å to the ligand this residue seems not to be directly involved in the catalysis.

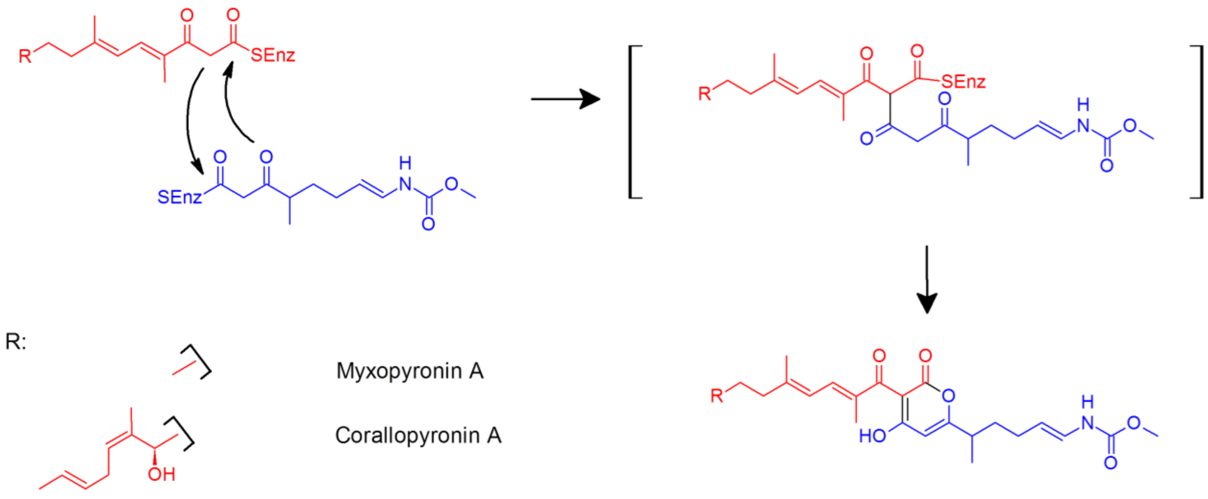

Figure S5: Last step of myxopyronin A [15] and corallopyronin A [16] biosynthesis. The western (red) and the east (blue) chains are thought to be condensed by the catalytic activity of the KS MxnB or CorB, respectively. The intermediate shown here follows from the nucleophilic attack of the deprotonated α-carbon of the western chain with the carbonyl carbon of the eastern chain. Both intermediates were used for docking studies.

```
PpyS INSRYWLADNEKPMELIEAAFNHALAQANIIKEDIDLLIYSSVARGFIEPA 107
YP42 LVNRRWCDSHESPIDHVAMATRKALSETYLRPEHIELFIYVGIGRGFLEPG 101
WP16 SDVRYWLDKDEKPIDLVARAFQEAINEANCDKDDIDLLVYTGVGRGFIEPA 102
PyrS SGTRHWLGANETPMELMETAFNSALAQANIDKADLDLLIYPNVTRGFIEPA 102
OleA IHARRLWDQDVQASDAATQAARKALIDANIGIEKIGLLINTSVSRDYLEPS 106
FabH IRERHIAAPNETVSTMGFEAATRAIEMAGIEKDQIGLIVVATTSATHAFPS 89
CorB IRERRWVKD-ETASFMGAEAAKEAVRDAGLKLEDIDLIINASGSPEQAVPD 96
MxnB IRERRWVKG-ETAAFMGAEAAKEAVRDAGLQLSDIDLIISASGSPQQAVPD 96
Cpz6 -VGRRNLTAFADACEMAVDAARRALAATGLVADDIDAIVTSH-TTSWTLPN 114
Gcs  TVQERTAPAWEAVQAYGERAARGALQIAGLDVADVDCLITSN-STTPALPG 174

PpyS NSTFVSKALGLT---CRNYDVVDACNGWVAAMDIINSKMKAGEIRHAAIVN 155
YP42 NSHMMASTLGFIN--AECFDVVDACMSWTRAMSIIDSLFKCGQYRNAMIVN 150
WP16 AAYHVAASLGLQN--AECFDILDACMSWTRVLNIVYSLFKSGRYKRALIVN 151
PyrS NSTFIAKALGLS---CRNFDVVDACNGWVTAMDVINSKMQAGEIRYAAIVN 150
OleA TASIVSGNLGVSD-HCMTFDVANACLAFINGMDIAARMLERGEIDYALVVD 156
FabH AACQIQSMLGIKG--CPAFDVAAACAGFTYALSVADQYVKSGAVKYALVVG 138
CorB GGPLVQRELGLGRSGVPSITVNASCLSFFVALDVAANYLNMRRYKRILIVS 147
MxnB GGPLVQRELGLGRSGTPAITVNASCLSFFVALEVASNYLNMRRYRRILVVS 147
Cpz6 LDVHLVEVLGLRPDVSRVALTSLACAGGTQALVRAADQLRARPGGKVLVVV 165
Gcs  LDVALANRLPLRGDTMLLPATQWACVAGTRSLALAADLVAADPDRVVLVVI 225

PpyS ...IEHIFIHTGSPKTWARLGQKMGID-DKIHHVGQR----VGNIATASIP 317
YP42 ...IDIVFTHASSKAAWHGYGEKVGIQ-DKMYHIYPE----TGNLVSASIP 309
WP16 ...IRAIFPHASSKREWDKVAEALNIK-HLLWHIYPT----YGNLVSASVP 161
PyrS ...VHKVFIHTGSPKMWEHIGQLIGID-HKLHHVGHK----TGNIITASIP 313
OleA ...LDQFVIHQVSRPHTAAFVKSFGIDPAKVMTIFGE----HGNIGPASVP 309
FabH ...LDWLVPHQANLRIISATAKKLGMSMDNVVVTLDR----HGNTSAASVP 281
CorB ...CRYVIPHQPSRVVLDYLS-LTYPD-DKLVRIIDR----FANCIGASMP 299
MxnB ...VKYIIPHQPSRVVLDYLS-LSYPE-EKLIRIIER----FGNCIGASMP 299
Cpz6 ...PEFAVVHPGGPRIISEVTAALGLDAARTRHSFAS-LEENGNLGGNAVL 307
Gcs  ...PDVLLAHPGGTRVLEYMEQTMPDEWPSGLLSYSRDSYTSGNRGGAAVF 377
```

Figure S6: A multiple sequence alignment (ClustalW, standard parameters) of PpyS from *P. luminescens* TT01, YP_004230959 (YP42) from *Burkholderia sp.* CCGE1001, WP_016949109 (WP16) from *Anabaena sp.* PCC 7108, WP_007967127 (PyrS) from *Pseudomonas sp.* GM30, OleA from *X. campestris*, FabH from *E. coli*, CorB from *C. coralloides*, MxnB from *M. fulvus*, Cpz6 [17] (caprazamycin biosynthesis) from *Streptomyces* sp. MK730-62F2 and Gcs [18] (germicidin synthase) from *S. coelicolor*. Highly conserved residues are shown in grey, conserved catalytic triad and position of E105 from PpyS are highlighted in black.

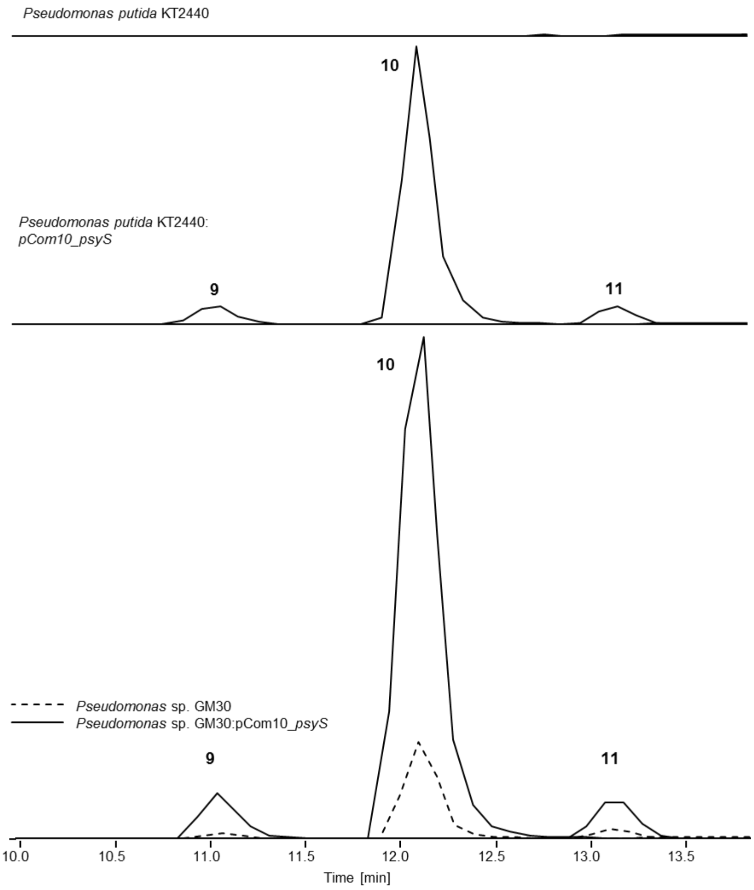

Figure S7: Extracted-ion chromatograms (EICs) of pseudopyronines. **9** (*m*/*z* 267.2 $[M + H]^+$), **10** (*m*/*z* 295.2 $[M + H]^+$), **11** (*m*/*z* 323.2 $[M + H]^+$). In *Pseudomonas putida* KT2440 no pseudopyronines could be detected (top chromatogram). All chromatograms are drawn to the same scale.

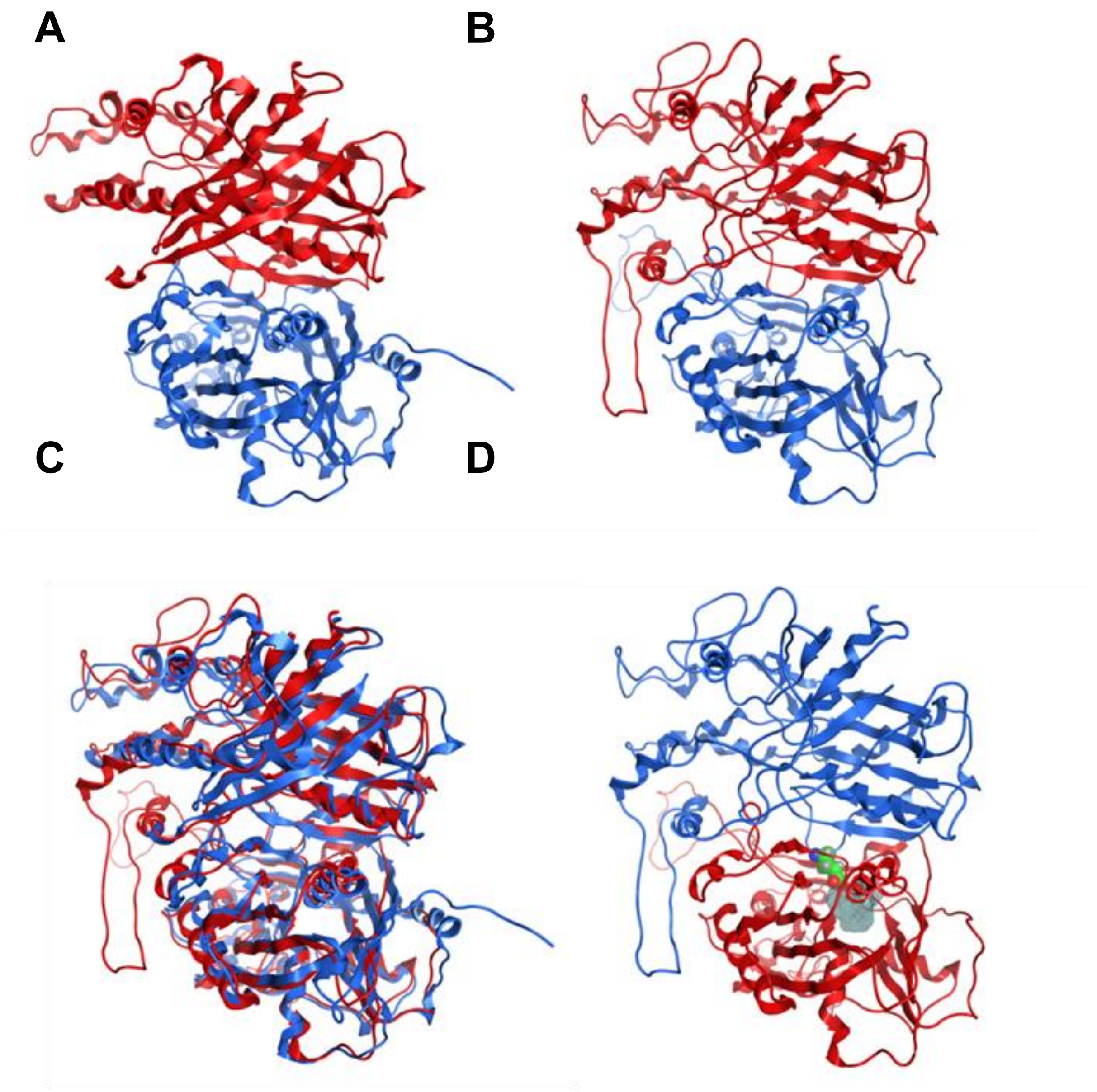

Figure S8: Structure of OleA-dimer (PDB: 3ROW) from *Xanthomonas campestris* (A). Modeled structure of pseudopyronine synthase (PyrS)-dimer from *Pseudomonas* sp. GM30 (B), which was generated using the OleA structure as a template. The superposition of OleA (blue) and PyrS (red) structures (C) revealed a root-mean-square deviation (RMSD) of 3.3 Å. The modeled PyrS-dimer structure with covalently to Cys124 docked pseudopyronine B intermediate (**19**, D). In a green sphere representation Glu100 is shown, which is the analogue to Glu105 of PpyS.

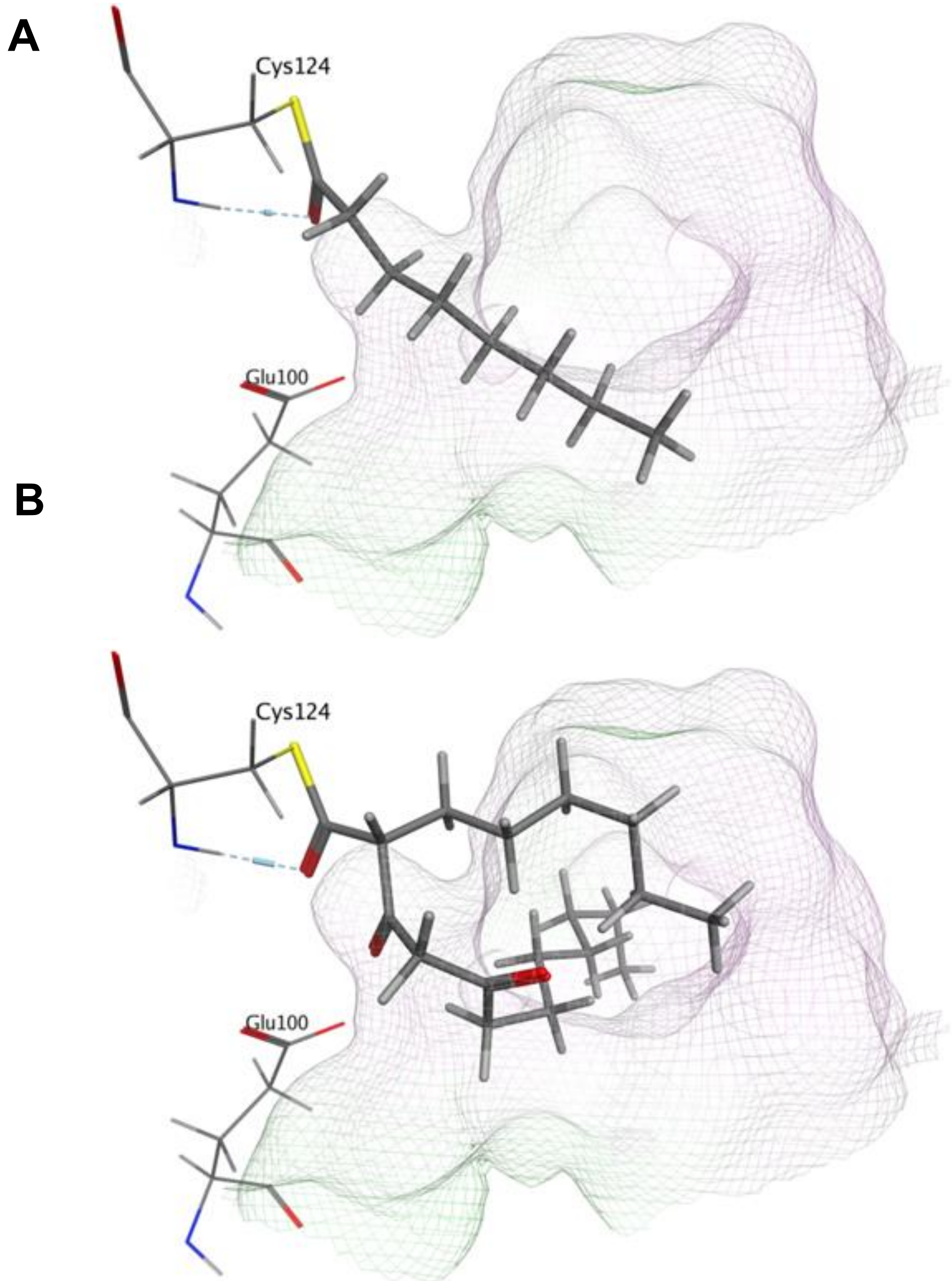

Figure S9: A detailed view of the proposed PyrS-binding pocket with covalently to Cys124 docked substrate (**17**, A) and intermediat (**19**, B) of pseudopyronine B. The catalytic triade consists of Cys124, His277 and Asn306. The cavity of the binding pocket is shown in a line representation, where green represents a lipophilic surface area, magenta a hydrophilic and white a neutral. Possible formed hydrogen bonds are shown as dashed blue lines.

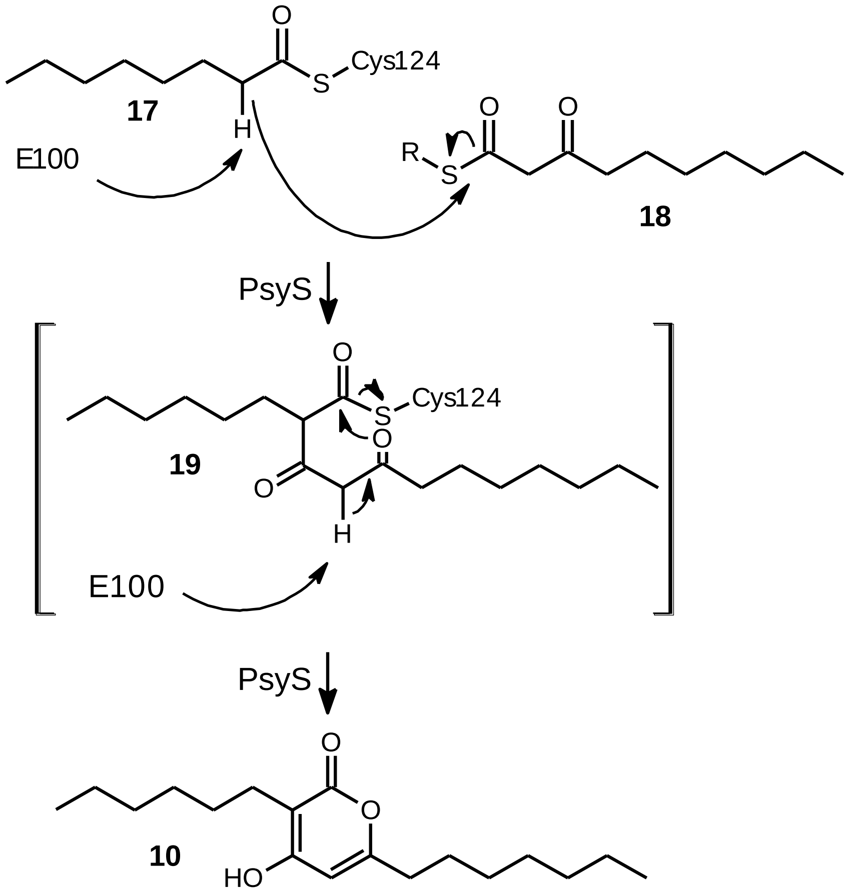

Scheme S1: Proposed biosynthesis of **10** by PyrS from *Pseudomonas* sp. GM30. In the first step octanoic acid (**17**) is covalently bound to the active site Cys124. The deprotonated α-carbon nucleophilicly attacks a 3-oxodecanoyl thioester (**18**, R = ACP or CoA) to form the covalently bound intermediate (**19**). Due to a spontaneous or a catalyzed deprotonation (by E100) the pyrone ring is formed and **10** is released from PyrS.

References

1. Adachi, Y.; Fukuhara, C. *Anal. Biochem.* **2012**, *431*, 66-68.

2. Brachmann, A. O.; Brameyer, S.; Kresovic, D.; Hitkova, I.; Kopp, Y.; Manske, C.; Schubert, K.; Bode, H. B.; Heermann, R. *Nat. Chem. Biol.* **2013**, *9*, 573-578.

3. Gibson, D. G.; Young, L.; Chuang, R.; Venter, J. C.; 3rd, C. A. H.; Smith, H. O. *Nat. Methods* **2009**, *6*, 343-345.

4. Altschul, S. F.; Gish, W.; Miller, W.; Myers, E. W.; Lipman, D. J. *J. Mol. Biol.* **1990**, *215*, 403-410.

5. Berman, H. M.; Westbrook, J.; Feng, Z.; Gilliland, G.; Bhat, T. N.; Weissig, H.; Shindyalov, I. N.; Bourne, P. E. *Nucleic Acids Res.* **2000**, *28*, 235-242.

6. Larkin, M. A.; Blackshields, G.; Brown, N. P.; Chenna, R.; McGettigan, P. A.; McWilliam, H.; Valentin, F.; Wallace, I. M.; Wilm, A.; Lopez, R.; Thompson, J. D.; Gibson, T. J.; Higgins, D. G. *Bioinformatics* **2007**, *23*, 2947-2948.

7. Jones, G.; Willett, P.; Glen, R. C.; Leach, A. R.; Taylor, R. *J. Mol. Biol.* **1997**, *267*, 727-748.

8. Korb, O.; Stützle, T.; Exner, T. E. *J. Chem. Inf. Model.* **2009**, *49*, 84-96

9. Guindon, S.; Gascuel, O. *Syst. Biol.* **2003**, *52*, 696-704

10. Hanahan, D. *J. Mol. Biol.* **1983**, *166*, 557-580.

11. Brown, S. D.; Utturkar, S. M.; Klingeman, D. M.; Johnson, C. M.; Martin, S. L.; Land, M. L.; Lu, T. S.; Schadt, C. W.; Doktycz, M. J.; Pelletier, D. A. *J. Bacteriol.* **2012**, *194*, 5991-5993

12. Nelson, K. E.; Weinel, C.; Paulsen, I. T.; Dodson, R. J.; Hilbert, H.; Santos, dos, V. A. P. M.; Fouts, D. E.; Gill, S. R.; Pop, M.; Holmes, M.; Brinkac, L.; Beanan, M.; DeBoy, R. T.; Daugherty, S.; Kolonay, J.; Madupu, R.; Nelson, W.; White, O.; Peterson, J.; Khouri, H.; Hance, I.; Lee, P. C.; Holtzapple, E.; Scanlan, D.; Tran, K.; Moazzez, A.; Utterback, T.; Rizzo, M.; Lee, K.; Kosack, D.; Moestl, D.; Wedler, H.; Lauber, J.; Stjepandic, D.; Hoheisel, J.; Straetz, M.; Heim, S.; Kiewitz, C.; Eisen, J. A.; Timmis, K. N.; Düsterhöft, A.; Tümmler, B.; Fraser, C. M. *Environ. Microbiol.* **2002**, *4*, 799-808.

13. Smits, T. H.; Seeger, M. A.; Witholt, B.; van Beilen, J. B. *Plasmid* **2001**, *46*, 16-24.

14. Proschak, A.; Zhou, Q.; Schöner, T.; Thanwisai, A.; Kresovic, D.; Dowling, A.; ffrench-Constant, R.; Proschak, E.; Bode, H. B. *ChemBioChem* **2014**, *15*, 369-372.

15. Sucipto, H.; Wenzel, S. C.; Müller, R. *ChemBioChem* **2013**, *14*, 1581-1589.

16. Erol, O.; Schäberle, T. F.; Schmitz, A.; Rachid, S.; Gurgui, C.; Omari, el, M.; Lohr, F.; Kehraus, S.; Piel, J.; Müller, R.; König, G. M. *ChemBioChem* **2010**, *11*, 1253-1265.

17. Tang, X.; Eitel, K.; Kaysser, L.; Kulik, A.; Grond, S.; Gust, B. *Nat. Chem. Biol.* **2013**, *9*, 610-615.

18. Chemler, J. A.; Buchholz, T. J.; Geders, T. W.; Akey, D. L.; Rath, C. M.; Chlipala, G. E.; Smith, J. L.; Sherman, D. H. *J. Am. Chem. Soc.* **2012**, *134*, 7359-7366.

^{1}H-NMR spectrum of pseudopyronine C, DMSO-*d6*

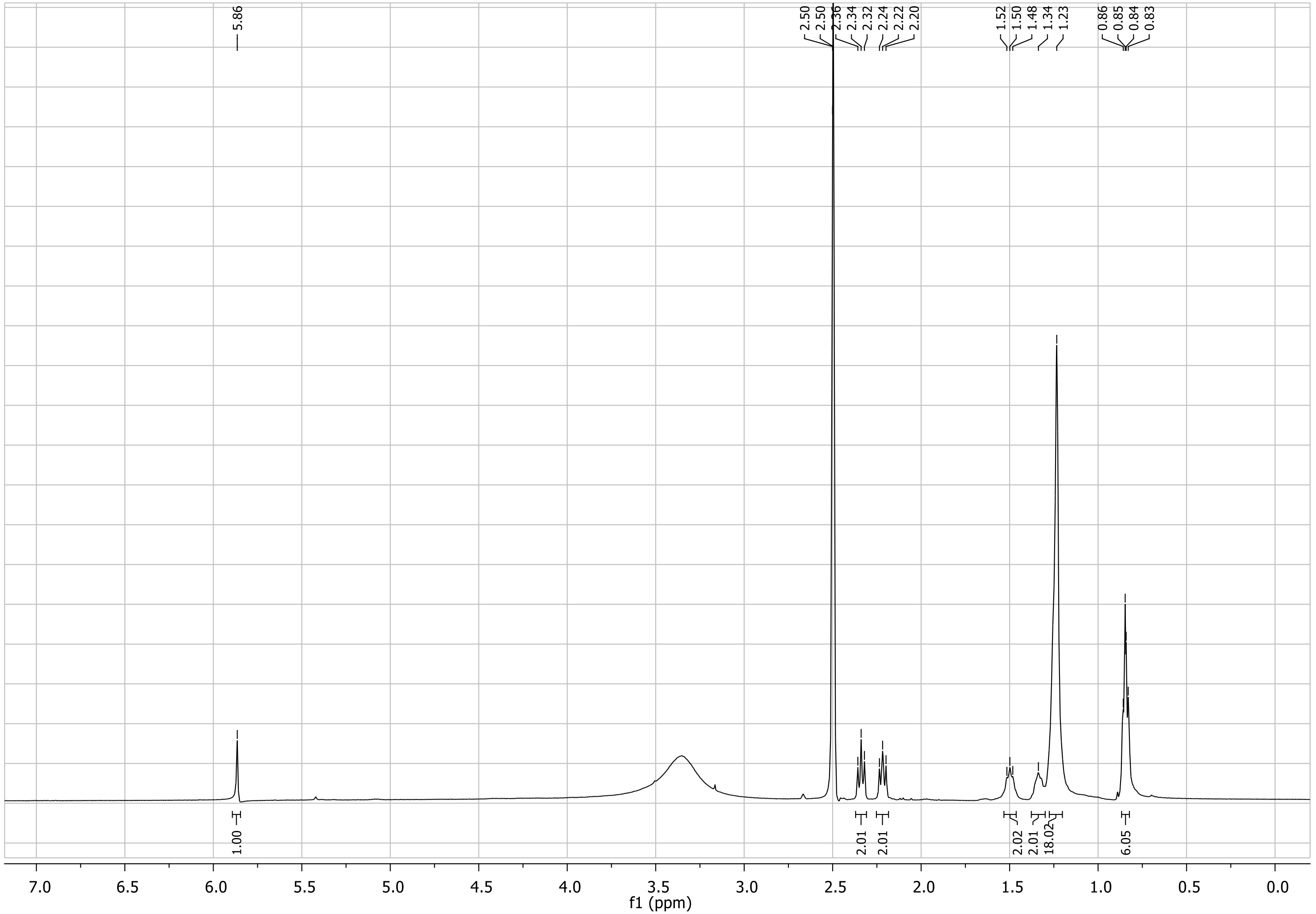

^{13}C -NMR spectrum of pseudopyronine C, DMSO-*d6*

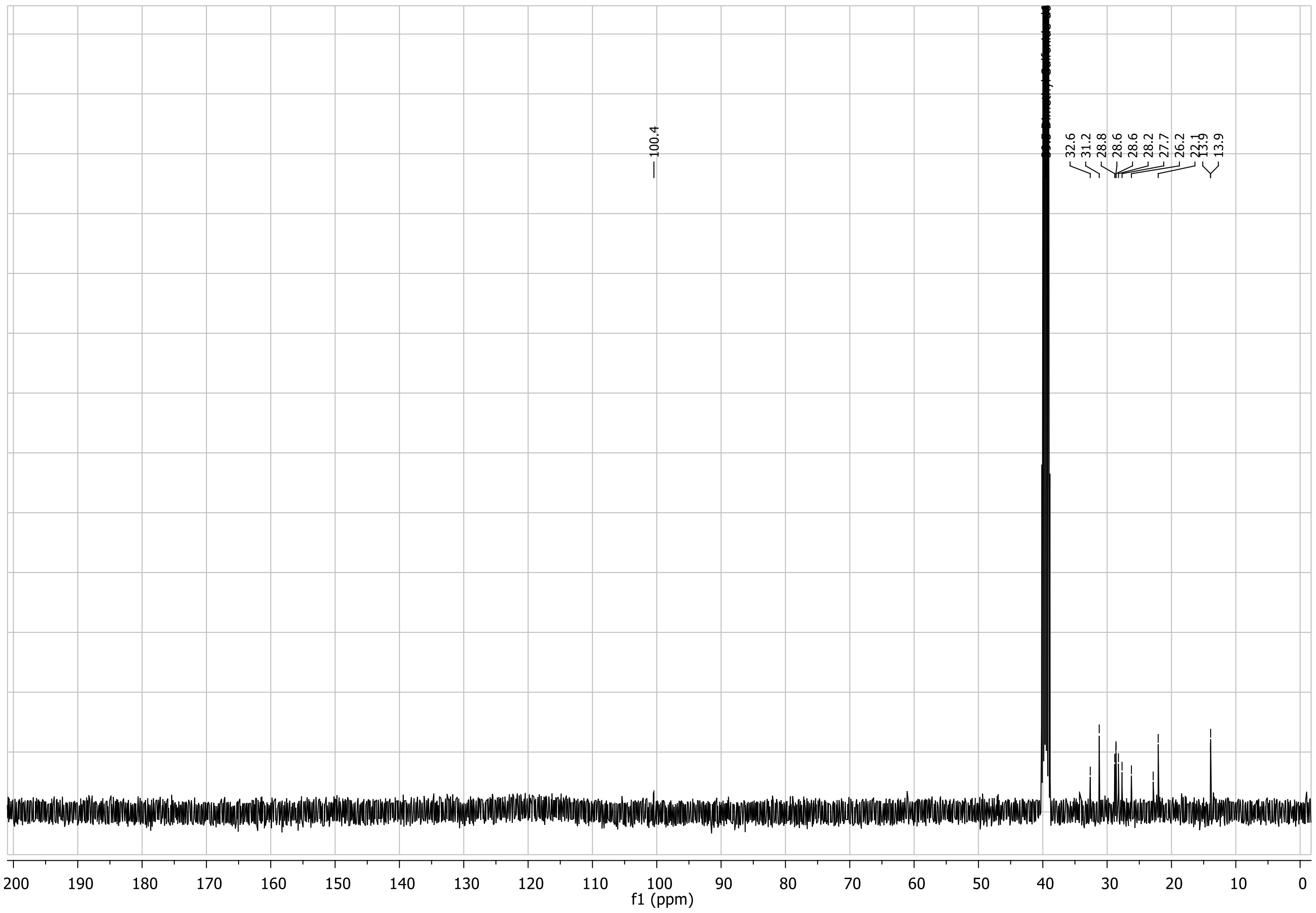

COSY spectrum of pseudopyronine C, DMSO-*d6*

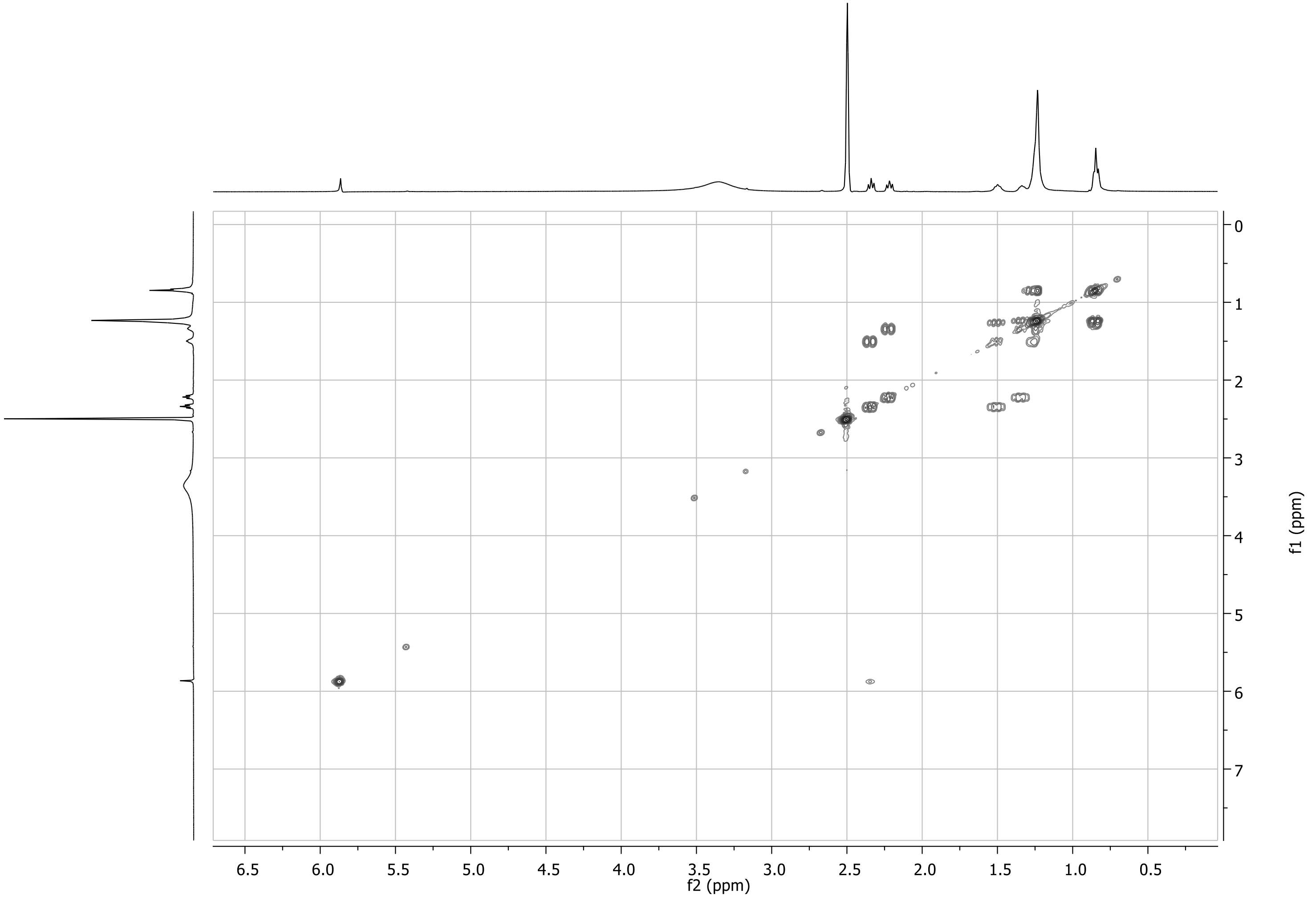

HSQC spectrum of pseudopyronine C, DMSO-*d6*

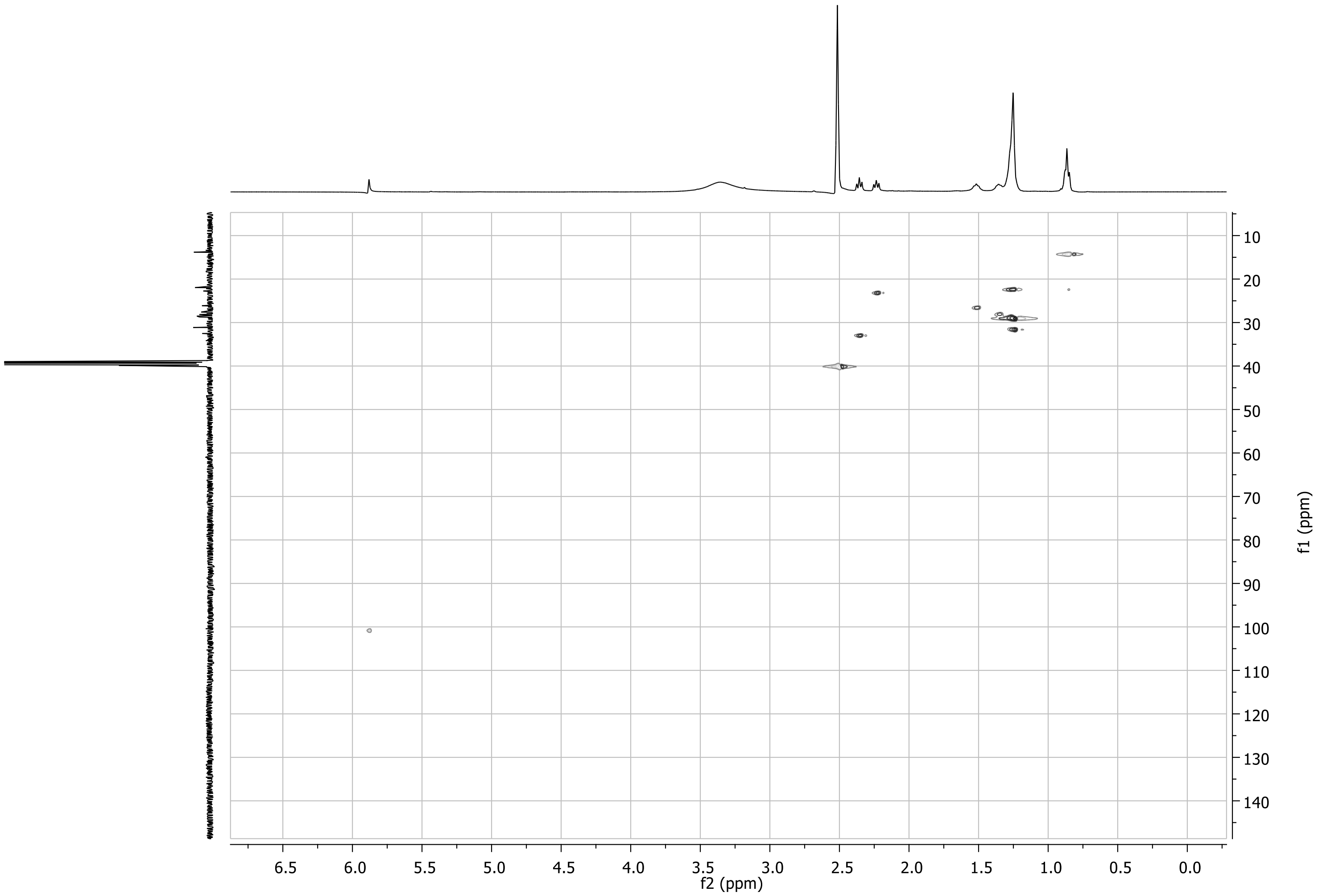

HMBC spectrum of pseudopyronine C, DMSO-*d6*

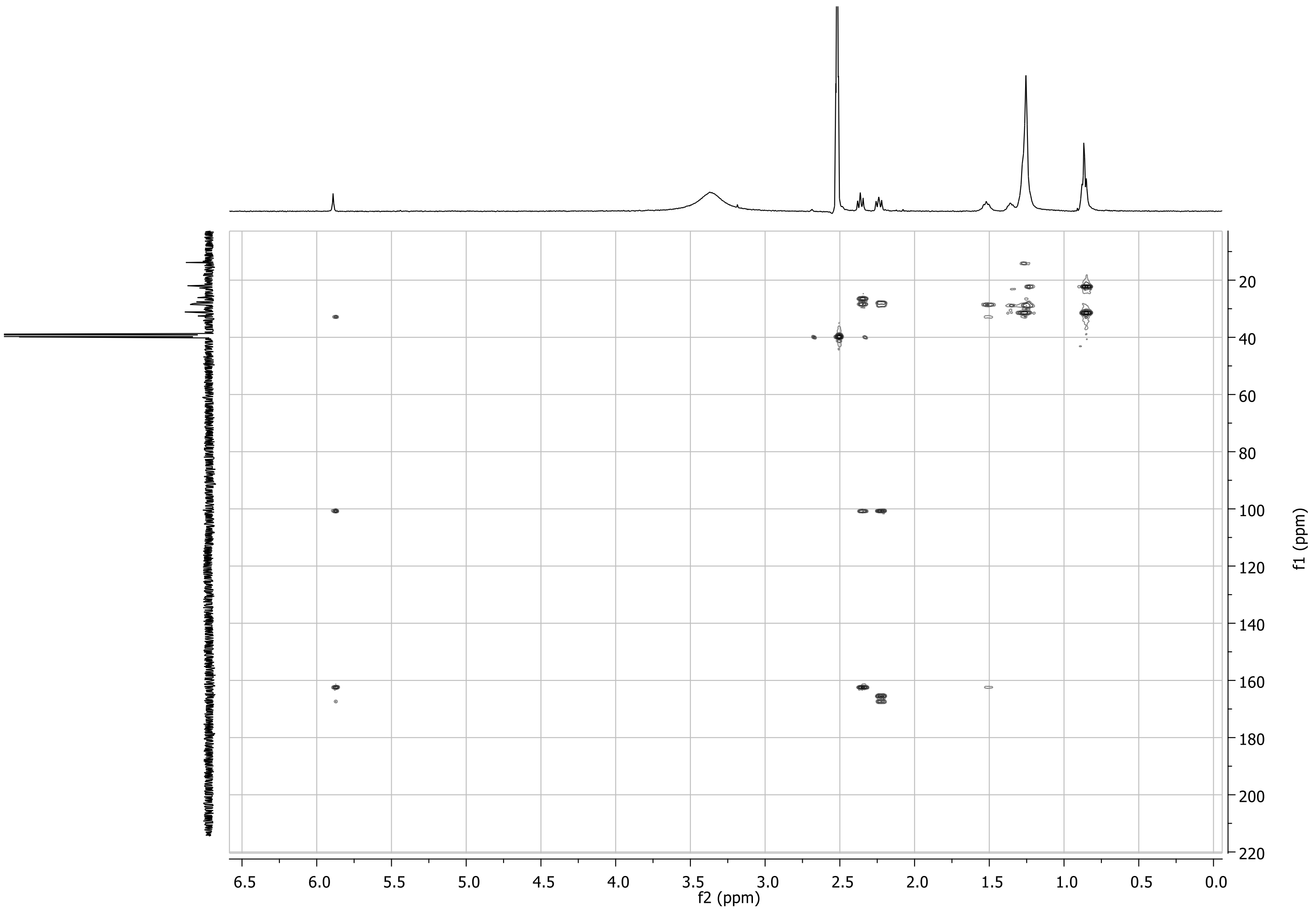

Supplement: File 1 — Experimental procedures, details of bioinformatic analysis and NMR data of pseudopyronines. [file Beilstein_J_Org_Chem-11-1412-s001.pdf]
